# “A” for Effort: Rewarding Effortful Retrieval Attempts Improves Learning From General Knowledge Errors in Women

**DOI:** 10.3389/fpsyg.2019.01179

**Published:** 2019-06-21

**Authors:** Damon Abraham, Kateri McRae, Jennifer A. Mangels

**Affiliations:** ^1^Department of Psychology, University of Denver, Denver, CO, United States; ^2^Department of Psychology, Baruch College and The Graduate Center, The City University of New York, New York, NY, United States

**Keywords:** reward, intrinsic, extrinsic, feedback, cognitive evaluation theory, testing effect, gender difference, mastery achievement goals

## Abstract

Previous research has shown that the prospect of attaining a reward can promote task-engagement, up-regulate attention toward reward-relevant information, and facilitate enhanced encoding of new information into declarative memory. However, past research on reward-based enhancement of declarative memory has focused primarily on paradigms in which rewards are contingent upon accurate responses. Yet, findings from test-enhanced learning show that making errors can also be useful for learning if those errors represent effortful retrieval attempts and are followed by corrective feedback. Here, we used a challenging general knowledge task to examine the effects of explicitly rewarding retrieval effort, defined as a semantically plausible answer to a question (referenced to a semantic knowledge database www.mangelslab.org/bknorms), regardless of response accuracy. In particular, we asked whether intermittent rewards following effortful incorrect responses facilitated learning from corrective feedback as measured by incidental learning outcomes on a 24–48 h delayed retest. Given that effort-contingent extrinsic rewards represent the intersection between an internal locus of control and competency, we compared participants in this “Effort” group to three other groups in a between-subjects design: a Luck group that framed rewards as related to participant-chosen lottery numbers (reward with internal control, not competence-based), a random Award group that framed rewards as computer generated (no control, not competence-based), and a Control group with no reward, but matched on all other task features. Both men and women in the Effort group showed increased self-reports of concentration and positive feelings following the receipt of rewards, as well as subjective effort on the retest, compared to the Control group. However, only women additionally exhibited performance benefits of effort framing on error correction. These benefits were found for both rewarded and non-rewarded trials, but only for correction of low confidence errors, suggesting that effort-contingent rewards produced task-level changes in motivation to learn less familiar information in women, rather than trial-level influences in encoding or consolidation. The Luck and Award groups did not demonstrate significant motivational or behavioral benefits for either gender. These results suggest that both reward context and gender are important factors contributing to the effectiveness of rewards as tools to enhance learning from errors.

## Introduction

“Learning is its own greatest reward.” This statement, credited to the humanistic British writer William Hazlitt (1778–1830), epitomizes a certain educational ideal, one that values intrinsic rewards from the effort put into mastering new material, regardless of the ultimate outcome measured by external metrics of achievement. Indeed, decades of social cognitive and educational research have shown that students who are motivated to master material fare better with regard to overall learning, particularly in the face of negative outcomes and setbacks, as compared to when motivation is focused more on a desire to prove one’s ability relative to others (Grolnick and Ryan, 1987; Henderson and Dweck, 1990). More specifically, within the context of learning new factual information, the willingness to engage effortfully with this material by making valid initial retrieval attempts, even when errors may occur, has been championed as superior to simply being told answers, or giving answers that have little semantic relationship to the question (e.g., testing-effects; error-based learning; Metcalfe, 2017; see also Kluger and DeNisi, 1998). Given the apparent importance of effortful engagement for learning, one question is whether providing extrinsic rewards related to perceived engagement, rather than outcome, can yield the same benefits for feedback-based learning that has been shown for intrinsic mastery-oriented mindsets (Mangels et al., 2006; Moser et al., 2011; Ochakovskaya, 2018). To this aim, the present study will focus on how rewarding students for high-quality, but nonetheless incorrect responses to a series of difficult general-knowledge questions may facilitate the ability to encode corrective information and learn from those mistakes.

Recent classroom field studies (Angrist et al., 2014; Levitt et al., 2016a, b) and controlled laboratory experiments (Murayama and Kuhbandner, 2011; Patil et al., 2016) have shown that the prospect of attaining a monetary reward can be a useful motivator for encoding new information in declarative memory, particularly when that encoding depends on forming new associations rather than simply memorizing individual items (Murty and Adcock, 2013; Stanek et al., 2019). Giving rewards for successful learning can promote overall task engagement, as well as up-regulate attention specifically to reward-relevant information (Wittmann et al., 2005). However, Cognitive Evaluation Theory underscores how the use of rewards to extrinsically motivate behavior oftentimes leads to a paradoxical decrease in intrinsic motivation, lessening the person’s natural inclinations to participate in the task at hand (Deci, 1975; for meta-analyses see Deci et al., 1999, 2001). Individuals who are rewarded for good performance may feel their behavior is coerced by the reward contingencies, and this can subvert their sense of autonomy and shift the perceived locus of control from internal to external factors (e.g., Lepper et al., 1973; Ryan, 1982; Ryan et al., 1983; Mueller and Dweck, 1998). Likewise, intrinsic motivation is negatively impacted when rewards are given just for “showing up” or for completion regardless of performance because these decrease the association of the reward with competence. Intrinsic motivation can be maintained or increased, however, when rewards are unexpected, are tied to an internal locus of control, and/or provide positive information about competence. Therefore, it is possible that intermittently rewarding individuals for their competence regarding task engagement and completion, regardless of performance outcomes — essentially rewarding their “effort” to make a high quality response in a task even if their attempt was unsuccessful — should be particularly beneficial to a difficult declarative memory task that might require sustained attention and/or where individuals might feel particularly challenged.

### Study Design

Our examination of the influence of an “effort-contingent” reward on feedback-based learning in declarative memory used a test-feedback-retest paradigm that has been extensively studied in our laboratory (Butterfield and Mangels, 2003; Mangels et al., 2011, 2017, 2018; Whiteman and Mangels, 2016). In this general paradigm, a trial begins with subjects generating an answer to a general knowledge question, selected to be challenging to that individual based on their knowledge level, and rating their confidence in their answer. Entering this information triggers presentation of feedback indicating what the correct answer actually is (which simultaneously confirms the accuracy or inaccuracy of their own retrieval attempt). Critically for the present study, this feedback was followed by a reward-related stimulus that indicated whether the trial additionally earned them a token toward lottery tickets that might earn them additional monetary prizes at the end of the study. Later, participants were given a surprise retest for all questions, both those initially correct and incorrect, allowing us to operationalize successful learning as the number of initially incorrect items that were successfully corrected on the retest. Reward-earning stimuli were presented occasionally (∼25–30% of all trials), but equally often after both correct and incorrect answers, and thus, are neither contingent on initial performance, nor (because it was a surprise retest) on later memory, unlike in many past studies (Adcock et al., 2006; Wittmann et al., 2011; but see Mather and Schoeke, 2011; Murty and Adcock, 2013). We were particularly interested in determining whether framing rewards as “effort-contingent” influenced error correction for either the rewarded incorrect trials specifically, or even incorrect trials on the whole, compared to experimental groups where rewards were not contingent on effort, or were not given at all.

Rewards in the Effort group were explicitly described to participants as contingent on the semantic quality of their answer in terms of semantic proximity to the question and correct answer, regardless of absolute accuracy. Thus, although there are multiple ways to operationalize the motivational construct of “effort” (e.g., Niv et al., 2006; Schunk et al., 2008), our definition focuses on quality of the response (i.e., whether a teacher or fellow student might consider that they “showed an effort” to provide an educated response, even if it was incorrect), rather than the quantity of physical or mental exertion needed by the participant to provide this higher-quality answer (e.g., Wolf et al., 1995; Wise and Kong, 2005; Manohar et al., 2017). We opted to focus on answer quality in part because our extensive past use of this general knowledge paradigm has allowed us to develop a large database of questions and common answers^1^ from which we can derive both the overall difficulty level of the question, as well as *a priori* judgments about the quality associated with participants’ incorrect responses (see section “Materials” for judgment criteria). Specifically, in addition to the correct answer, only those incorrect responses that matched a semantically plausible incorrect response in the database were tagged as eligible for reward. Additionally, we biased rewards toward the more difficult items in our general knowledge question pool. We acknowledge that high-quality answers for questions with high overall accuracy rates (i.e., “easier” questions), may have high retrieval fluency and can be answered quickly with little retrieval effort on the part of the participant. On the other hand, more difficult questions typically have a larger range of possible answers, which increases retrieval difficulty. Thus, it is these more difficult questions, where initial answers might be associated with low confidence, where we expected that the prospect of reward for an educated guess might yield the greatest benefits to learners.

We attempted to isolate the mechanism underlying any observed effects of the Effort group on learning by contrasting it with two additional reward groups (Luck and Award), as well as a non-reward control group. In the Luck group, participants selected a set of “lucky numbers” at the outset of the experiment and were told that a reward would be delivered if one of these numbers matched a computer-generated number on a given trial (see also Wittig et al., 1981; Schunk, 1985). These participants, therefore, had some degree of internal control, although the rewards did not provide any competency information. In the Award group, participants were told that rewards would occur randomly on computer-selected trials. Thus, while rewards were presented, they did not speak to the participants’ competency for the task, and by being attributed to a random event, the locus of control was shifted away from the individual. Finally, we introduced a Control group to address the basic impact of the novelty and perceptual salience of the reward stimulus on memory (Horvitz, 2000; Bunzeck et al., 2010). Here, the same stimuli that signified rewards in the other groups were framed simply as randomly appearing tokens. To ensure that participants still attended to the appearance of these items, however, they were told they would be rewarded at the end of the task for maintaining an accurate count of these tokens (all participants were asked to provide a token count at the end of a block of trials, but only this group was rewarded for count accuracy). The actual reward contingencies were equated across groups not only with regard to quantity (∼25–30% of all trials), but also with regard to the quality of the participants’ answer attempt and general task effort. Only the instructional framing of the contingencies on which these post-feedback stimuli appeared was manipulated across groups.

### Study Hypotheses and Predictions

We predicted that framing rewards as effort-contingent would result in increased intrinsic motivation to attend to the task and in particular, to the corrective feedback, compared to the Control group and the two non-competency-contingent reward groups (Luck and Award). This would be evidenced by better incidental learning of the correct answers, as revealed by performance on the surprise retest, as well as higher self-reported task-motivation and concentration. Additionally, as discussed in greater detail below, the effort-contingent framing may affect encoding task-wide or might be more specific only to those question-answer trials that were rewarded, especially those with lower initial response confidence because these require more externally motivated effort for learning. We also explore whether the influence of effort-contingent reward in a verbal declarative memory task, such as used here, may vary across individuals on the basis of gender and motivational predispositions.

As mentioned above, the effects of our reward manipulations on successful incidental encoding of the correct answer might be evidenced task-wide, affecting both rewarded and non-rewarded trials, or might be greater for those trials that received a reward. To the extent that the prospect of reward promotes overall attention and task-engagement (i.e., proactive control; Locke and Braver, 2008; Chiew and Braver, 2013, 2016; but see Kostandyan et al., 2019), perhaps through tonic increases in dopamine levels (Floresco et al., 2003; Lisman and Grace, 2005), we might expect group differences in retest performance to be generalized across all trial types. However, most reward paradigms, such as the monetary incentive delay task, signal the possibility for reward prior to the beginning of each trial (e.g., Knutson et al., 2000; Adcock et al., 2006; for review see Krebs and Woldorff, 2017), thereby additionally potentiating transient levels of effort at the trial-level. Additionally, the reward presentation itself should elicit phasic increases in dopamine (Schultz, 1998, 2001; Glimcher, 2011). In our task the reward is presented after the memory-relevant information, making it is less clear how this phasic increase might influence learning. Yet, updated reward learning models suggest a potential “penumbra” effect by which memory for other information presented within the same context might similarly be enhanced despite being somewhat unrelated to and temporally separated from with the reward itself (Wang et al., 2010; Lisman et al., 2011; Redondo and Morris, 2011). Indeed, Murayama and Kitagami (2014) recently demonstrated a reward memory enhancement effect for information that was not only presented before the reward stimulus but was also part of a separate task (Murayama and Kitagami, 2014). Thus, it is possible that there will an additional memory enhancement specific to rewarded trials.

We also considered how the confidence level the participants had in their initial answer might interact with reward enhancement. In previous studies with a similar paradigm, when participants provide an incorrect answer with high confidence, they are more likely to correct these items on the retest than if they endorsed their answer with low confidence (Butterfield and Metcalfe, 2001, 2006; Butterfield and Mangels, 2003). This effect is due in part to the increase in attention associated with the surprise of receiving negative performance feedback (i.e., finding out they are wrong equates to a large negative prediction error; see Butterfield and Mangels, 2003; Butterfield and Metcalfe, 2006), and in part to the greater likelihood that the participant will be familiar with the correct answer and the associated semantic facilitation that this affords (Butterfield and Mangels, 2003; Metcalfe and Finn, 2011; Sitzman et al., 2015). To the extent that correction of high-confidence errors is already strongly facilitated by these mechanisms, it may benefit little from the addition of the reward context in comparison to low-confidence errors. Additionally, low confidence errors may be more likely to occur in domains that are of less intrinsic interest to the participant, leaving them more malleable to the influence of extrinsic rewards (Kang et al., 2009; Gruber et al., 2014). For example, Murayama and Kuhbandner (2011) demonstrated reward-related memory enhancement for information that participants classified as “boring,” but not for information they classified as interesting. Therefore, we predicted that the general effect of reward context on overall performance should be most apparent for trials endorsed with low confidence.

Finally, the meaning and significance of a reward may potentially vary across individuals on the basis of gender and motivational predispositions (Shim and Ryan, 2005; Spreckelmeyer et al., 2009; Martin-Soelch et al., 2011). Women tend to exhibit better self-concepts than men in verbal domains (Skaalvik and Skaalvik, 2004; for meta-analyses see Wilgenbusch and Merrell, 1999; Huang, 2012) and self-concepts such as perceived competence are predictive of academic success (Miserandino, 1996). Moreover, some research suggests that women have greater levels of intrinsic motivation for academic learning more generally (Vallerand et al., 1992; but see Cokley et al., 2001). Moreover, women frequently outperform men in verbal and episodic memory tasks (Herlitz et al., 1997; Lewin et al., 2001; for a review, see Herlitz and Rehnman, 2008). Previous work in our lab has been consistent with these findings. In two prior studies using a similar paradigm to the present investigation, women tended to underperform relative to men on the first test, which primarily taps semantic memory, but outperformed men on error correction at the surprise retest, which relies heavily on episodic memory for new associations (Whiteman and Mangels, 2016; Mangels et al., 2018). Thus, women, who may be inherently more motivated to engage with learning this verbal information, might respond differently than men to a reward frame that is focused on effort. Therefore, our analyses considered gender differences in retest performance and looked for possible gender-by-group interaction effects as well.

## Materials and Methods

### General Design Overview

In a between-subjects, test-feedback-surprise-retest design, participants in four groups each answered 160 general knowledge questions. Question difficulty was titrated to achieve 50% accuracy at the first test. In all 4 groups, participants reported their confidence after each response, which was followed by the correct answer. Roughly 30% of responses were rewarded, evenly split between correct and incorrect trials. The framing of reward contingencies was manipulated for each group: trial-specific reward stimuli either indicated that the answer was deemed a “good effort” (Effort Group), that the program has drawn one of the participants’ lucky numbers chosen prior to the task (Luck Group), or that the program has randomly selected the question for reward (Award Group). In all conditions, the number of rewards received would provide additional opportunities for post-test compensation. For the Control group, there were no trial-specific rewards, but participants were told that maintaining an accurate count of the designated target symbol would provide opportunity for additional post-test compensation (Control Group). Following each block of 40 questions, participants self-reported level of effort and various measures of affect as well as how many rewards they had received in the previous block of trials. A surprise re-test of all 160 questions was conducted 1–2 days later, without reward.

### Participants

One hundred and sixty adults (101 women) were recruited from the University of Denver population and the surrounding community. Our target sample size of 140 participants (35/group) was based on prior studies we had conducted with this test-retest paradigm and other motivational and/or gender variables, where 20–35 participants were needed to achieve a significant group difference in error correction performance (Mangels et al., 2006, 2018; Ochakovskaya, 2018). However, based on our previous studies using a similar test-retest paradigm (Mangels et al., 2006; Whiteman and Mangels, 2016; Ochakovskaya, 2018), recruitment exceeded our target by ∼15% with the anticipation that some data would be unusable due to either attrition, technical problems, or titration cutoffs etc.

Participants were 18–35 years of age (*M* = 21.06, *SEM* = 0.021), native English speakers or fluent by 6-years-old and had normal or corrected to normal vision and hearing. Participants were randomly assigned to one of four study groups including three rewarded groups (Effort, Luck, and Award) and a control group. This study was carried out at the University of Denver in accordance with the recommendations of the University of Denver Institutional Review Board (DU IRB). The protocol was approved by the DU IRB prior to participant recruitment. All subjects provided written informed consent in accordance with the Declaration of Helsinki and were compensated at a rate of either $10 per hour or course credit.

Data from thirteen participants were lost due either to failure to complete the task (*N* = 1) or computer problems (*N* = 12). An additional three participants were excluded for failure to meet the titration target of 0.50 correct on the first test (performance <= 2 *SD* the sample mean; *M* = 0.4988, *SD* = 0.01). We removed four additional participants (1 from Control, 1 from Effort, and 2 from Luck) who were outliers based on having studentized deleted residuals of greater than |2| and/or Cook’s distance values of greater than 0.0277 (i.e., 4/sample size) on the second test.

Table 1 shows basic demographic information (gender, age, education) of the final sample. We did not specifically attempt to assign equal numbers of women and men to each group, but rather randomly assigned students to group regardless of gender. Despite more women than men participating overall, a non-significant Pearson chi-square test of independence verified that the gender ratios did not differ across groups, *X*^2^(3, *N* = 140) = 2.67, *p* = 0.45. There were no gender or group differences in participant age or years of education (all *p*s > 0.2).

**TABLE 1:** Sample characteristics.

	***n***	**Men**	**Women**	**Age men**	**Age women**	**Yrs. school men**	**Yrs. school women**
Control	35	12	23	20.33 (1.66)	21.52 (3.36)	15.42 (1.42)	14.89 (1.58)
Effort	35	15	20	20.87 (3.02)	20.70 (2.82)	15.13 (1.36)	15.40 (1.52)
Luck	34	15	19	21.20 (2.94)	20.53 (1.53)	15.57 (1.7)	14.87 (1.53)
Award	36	10	26	21.70 (2.81)	21.42 (2.4)	15.45 (1.39)	15.40 (1.73)

*Standard deviations appear in parentheses.*

### Materials

The task utilized 414 questions from the B-KNorms database of general knowledge questions and responses^2^. The questions in this database span a range of academic domains (history, literature, geography, etc.), and each question has a unique correct answer consisting of a single word, 3–12 characters long. The number of respondents to each question varied, but was 211 on average (min = 63, max = 583). The proportion of respondents giving the correct answer to a question provides a normative accuracy score (average accuracy across all database questions = 0.33). Additionally, the database provides a full list of all unique incorrect responses to each question (mean = 30 incorrect responses, min = 5, max = 79), as well as the proportion of responders who gave each of these unique incorrect responses. These incorrect answers varied in the degree to which they matched the correct answer and/or keywords in the question.

For the purpose of this study, the reward eligibility of answers in this database were rated by five independent raters, who based their judgment on whether the answer was both consistent with the content category of the question and constituted a quality response. By default, all correct responses were rated as reward-eligible. For an incorrect answer to be categorized as reward-eligible, however, it had to be rated as such by a consensus of 4–5 reviewers. Answers receiving reward-eligible votes by 3 or fewer raters were categorized as reward-ineligible. For example, if a subject answered a question asking for the name of an animal with “red,” this would not be considered a reward-eligible response. In some cases, an answer that otherwise matched the question keywords/correct response may still not be considered of sufficient quality to be reward-eligible. For example, “Smith” is a valid answer to any question asking for a proper name, however, because it is so common, is also unlikely to be a valid attempt to answer the question.

Additionally, although answers to all questions in the database were rated for reward-eligibility in this manner, at the task level, rewards/tokens were only presented on a subset of the more difficult questions. Rewards were not administered for the easiest quartile of the database (i.e., 104 easiest questions) regardless of whether a correct or reward-eligible answer was given. Exclusion of the easiest questions from reward eligibility served to support the perception of relationship between rewards and retrieval effort. The more difficult questions also had a larger base of incorrect answers that were rated as eligible for reward; questions with high accuracy rates had correspondingly few incorrect answers in the database.

### Design and Procedure

#### Overall Study Design

We employed a 2-day test-retest design with a delay interval of 1 or 2 days, balancing availability and delay intervals across groups. Prior to the start of the first test on Day 1, participants completed a series of computerized pre-test questionnaires consisting of demographic questions, followed by validated motivation questionnaires (e.g., Work Preference Inventory, Achievement Goals Questionnaire; see Supplementary Section “S1. Questionnaires”). Then they were randomly assigned to one of the four groups (Control, Award, Luck, Effort) and presented with 160 questions across 4 blocks of 40 questions each. At the end of each block, participants reported the number of reward stimuli they had seen (via free-response text box) and provided ratings of self-reported concentration, motivation, perceived difficulty and performance, and affective appraisals of the accuracy and reward feedbacks using a 7-point Likert scale. They also completed a motivation questionnaire at the end of the first test (see Supplementary Section “S1. Questionnaires”).

Throughout the task, the testing program selected questions based on an algorithm designed to titrate initial performance toward a cumulative accuracy level of 0.50. Titration was employed in order to equate overall accuracy and reward frequencies for each subject regardless of group. A target of 0.50 correct was selected as it provided an equal number of correct and incorrect responses for analysis and ensured that participants found the task challenging overall.

After attempting to answer each question, a series of feedback and reward outcomes were presented (see section “Trial sequence” for details). The adaptive computer program attempted to distribute rewards equally across the four test blocks, and within block, equally across correct and incorrect responses, while limiting rewards from appearing twice in a row. The target reward schedule was 12 rewards per block (6 correct, 6 incorrect), for a total of 24 correct and 24 incorrect rewarded trials across the task, which was equivalent to a 0.30 probability of reward. Provided the participant’s response was reward-eligible, the testing program attempted to reach this target reward schedule by applying an algorithm that iteratively adapted the probability of a reward based on the accuracy of the response and the total number of rewards previously administered in the given block of questions.

On Day 2 of testing, participants were given a surprise retest of all of the questions from Day 1. The block order was preserved from the first test, but question order within block was randomized. The second test contained no titration (performance was free to vary), nor reward feedback, nor post-block questions. At the conclusion of the retest, participants read a debriefing statement and then completed a short series of debriefing questions which asked about the participants’ perceived difficulty, level of effort, performance relative to others and degree to which the participants’ expected and/or studied for the surprise retest on a 9-point Likert scale. We then conducted a lottery for each participant and awarded any winning participants with money ($5, $15, and/or $25) in addition to their regular compensation for participation (see Supplementary Section “S2. Lottery Parameters” for details of lottery parameters).

#### Trial Sequence

**FIGURE 1:**
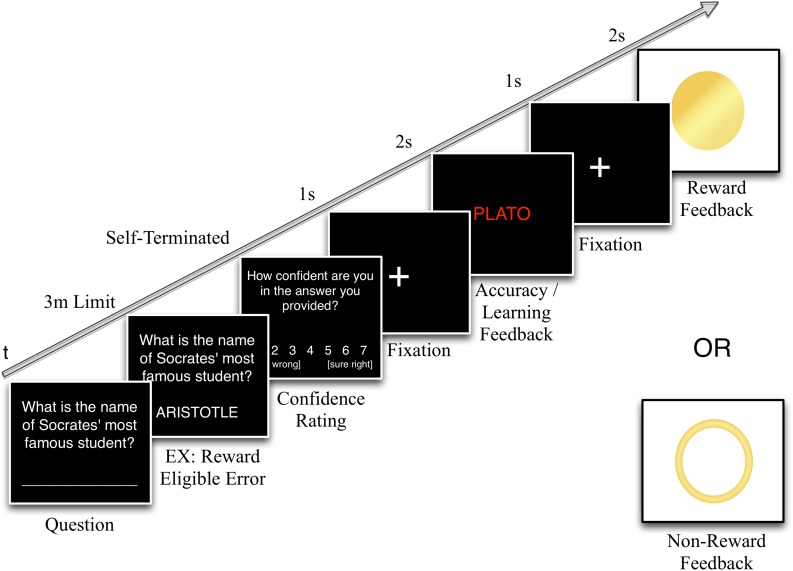
First-test trial structure. At the start of each trial, the participant is shown a question and types their answer in a blank space below. In this example, the answer entered is ARISTOTLE, which is incorrect, but reward-eligible because it is a plausible, semantically-relevant response. After the answer is entered, the participant would be prompted to rate their confidence in their answer’s accuracy on a 7-point scale (1 = sure wrong, 7 = sure right). Then, after a 1 s fixation point to orient attention to the center of the screen, the correct answer would appear for 2 s, either in red if their initial answer was incorrect, as in this example, or in green if it was correct. This accuracy/learning feedback is then followed by another 1 s fixation. Then, because this example shows a reward-eligible answer, the final stimulus would be either reward or no-reward feedback, assuming that this trial occurred in any of the three rewarded groups (in the Control group these stimuli function as target or non-target symbols to be counted). Both of these reward options would also have been possible if the participant had initially provided the correct answer (i.e., PLATO). However, if the participant had given an incorrect response that showed little effort and thus, was reward ineligible (e.g., SMITH or IDK), only non-reward feedback would have been possible as the final trial stimulus.

For each question, participants had 3 min to type and submit their one-word response (see Figure 1). A spell-checker was used to assist participants in correcting any misspelled words before they made their final response submission (see Supplementary Section “S3. Participant Response Spelling Check” for spell-checker rules). This was done to facilitate automated matching of the subject’s answers with the database answers for subsequent determination of reward eligibility by the program. Except for trials where no answer was given within the 3-min time limit (i.e., omit trials), the participant then rated their confidence in their response on a 7-point scale ranging from 1 (“sure wrong”) to 7 (“sure right”) immediately following each question. Omit trials skipped the confidence rating and went directly to feedback presentation. The testing program then compared the participant’s response to the correct answer to determine what type of accuracy feedback to provide (i.e., negative or positive feedback).

If the response was reward-eligible (see section “Materials” for how eligibility was defined), the program would then determine whether to provide a reward based on past reward history with the goal of keeping overall reward probability at 0.30, with an equal probability following both correct and incorrect answers. Responses for which no match could be found in the database were automatically ineligible for reward. Although the database is fairly comprehensive, we cannot rule out the possibility that some semantically plausible incorrect answers would not be found in the database and thus, deemed ineligible for reward on that basis rather than because they were poor matches for the question entity. However, not all reward-eligible responses in the database were rewarded either, so this should not have changed the subjective experience of the participant.

The feedback sequence began with a 1 s fixation crosshair followed by the accuracy feedback in which the correct answer was displayed for 2 s (green text / high tone for correct answers, red text / low tone for incorrect answers). Following another 1 s fixation, participants were presented with reward feedback. In order to control for the perceptual salience of the reward, two symbols (counterbalanced across participants) were used for both reward and non-reward feedback. Regardless of symbol type (open or closed circle), rewards were always coupled with a higher frequency tone and non-rewards with a lower frequency tone.

### Instructions

#### General Instructions

Pre-recorded instructions indicated that the trivia game was designed to be challenging and would adjust the difficulty of questions based on performance on prior questions but did not inform participants of the 0.50 titration target. Participants were shown sample questions and given examples of low-quality (i.e., reward ineligible) and high-quality (i.e., reward eligible) answers. The instructions for all groups except the control also indicated that the program would occasionally give rewards in the form of lottery tickets and were shown examples of the reward feedback. To attempt to equate the reward groups in terms of general motivation, all four instructions emphasized that reward opportunities would occur more often for questions that “exceed a certain level of difficulty.” In the Control group, they were told that the target stimulus would occur more often for these more difficult questions.

#### Group Framing Instructions

The specific instructions varied across the four groups in the degree to which they suggested participants had control over the reward outcomes, even though across all groups the testing program behaved identically in terms of reward frequency and eligibility criteria.

In the Effort group, the instructions emphasized that participants would be more likely to earn rewards when they made a “good effort” to provide a correct response. In the Luck group, participants selected 25 “lucky numbers” before the test using a Keno-style board showing numbers up to 100 and were told that the reward symbols appeared when the computer had randomly drawn one of their “lucky numbers.” In the Award group, participants were told that reward symbols appeared when the question number matched a number “randomly generated by the computer.” Incorrect answers could still be followed by a reward; however, they were told that the random number generator would only be triggered on more difficult questions (see Supplementary Section “S4. Group-Specific Instructions” for verbatim instructions).

Finally, in the Control group, participants counted the number of target stimuli (whichever was more infrequent) and reported this number at the end of each block. They were told that “the closer your answer is to the actual number displayed, the more lottery tickets you will be given.” As with the other groups, the target stimulus could follow both incorrect and correct responses and was more likely to occur for more difficult questions. As a manipulation check, we expected that participants in the Control group would maintain more accurate reward stimulus counts, given that they were directly incentivized to do so, and this was indeed the case (see Supplementary Section “S5. Post-block Reward Stimulus Count”).

### Data Analytic Strategy

Our primary behavioral measures were overall retest performance and error correction at retest. For each behavioral measure, we conducted a separate analysis of variance (ANOVA) including both group and gender as between-subject factors. In terms of error correction, we further explored trial-level learning performance with separate ANOVAs for rewarded versus non-rewarded items and for high versus low confidence items. For the confidence analysis, we first conducted a repeated-measures ANOVA with the confidence included as a within-subjects factor and group and gender as between-subjects factors.

For post-block and debriefing questions, we averaged responses for each question across blocks and conducted a 4 (group) by 2 (gender) multiple analysis of variance (MANOVA). Rank transformations were performed prior to the analysis as the distributions of some responses were sufficiently non-normal (skewness or kurtosis > |3|). Each subscale measure was included as a separate dependent variable. For the post-block questions and debriefing questions, each separate question was treated as a unique dependent variable.

Across each of these analyses, we set the criterion for significance as the conventional alpha level of *p* = 0.05. Main effects or interactions with an alpha level greater than 0.05 but less than 0.1 were considered marginal, but explored and reported as trends. Significant and marginal effects were further investigated by carrying out *post hoc* tests using the Bonferroni procedure for corrections for multiple comparisons.

## Results

### Retest Performance and Error Correction

#### Overall Performance

Prior to conducting these analyses, we established that first-test factors (first-test accuracy, the number of hours of delay between the first and second tests, the total number of reward/target stimuli shown, and the average confidence for rewarded and non-rewarded trials as a function of group and gender) that might influence retest performance had been successfully equated across groups and genders (see Supplementary Section “S6. First-Test Measures”).

**FIGURE 2:**
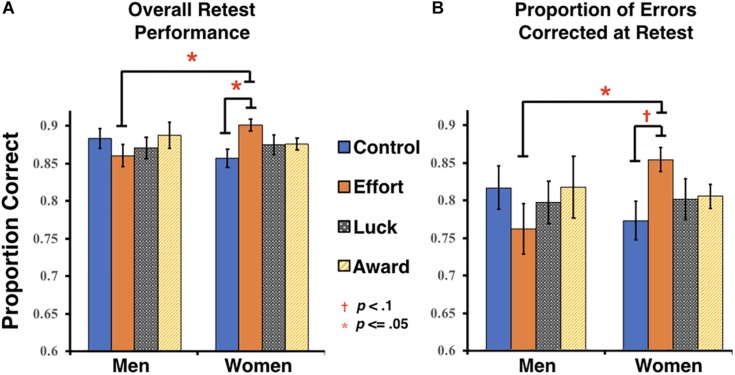
Retest performance measures. **(A)** Overall accuracy at retest (includes all first-test items, regardless of initial accuracy). **(B)** Proportion of first-test errors that were corrected at retest. Error bars represent the standard error of the mean (SEM).

The predicted main effect of group on overall retest performance was not significant, nor was there a main effect of gender (all *F*s < 0.41, *p*s > 0.74, η*p*^2^s < 0.01). However, there was a significant interaction between group and gender, *F*(3, 132) = 2.68, *p* = 0.05, η*p*^2^ = 0.057. *Post hoc* comparisons indicated that women in the Effort group (*M* = 0.90, *SD* = 0.04) had significantly higher overall retest scores than men in the same group (*M* = 0.86, *SD* = 0.06, *p* = 0.02), as well as women in the Control group (*M* = 0.86, *SD* = 0.06, *p* = 0.029) (see Figure 2A). No other comparisons between groups were significant in men or women.

An analysis focusing on the error correction rates followed a similar pattern. Neither of the main effects of group nor gender on the proportion of errors corrected were significant (all *F*s < 0.31, *p*s > 0.57, η*p*^2^s < 0.01), however, there was a marginally significant interaction between group and gender, *F*(3, 132) = 2.5, *p* = 0.063, η*p*^2^ < 0.054. *Post hoc* comparisons indicated that women in the Effort group (*M* = 0.81, *SD* = 0.06) corrected a significantly higher proportion of errors at retest than men in the same group (*M* = 0.74, *SD* = 0.11, *p* = 0.012) and corrected a marginally higher proportion than women in the Control group (*M* = 0.75, *SD* = 0.10, *p* = 0.083) (see Figure 2B).

Unfortunately, our samples were unmatched along the dimension of gender, as women outnumbered men across all groups. Therefore, the interaction effects were somewhat underpowered (observed power = 0.64 for overall retest performance and = 0.61 for proportion of errors corrected). In order to affirm that the lack of an observed effect in men was not due to an insufficient number of male participants, we estimated Bayesian factors for each null hypothesis using JASP software (JASP Team, 2018). A Bayesian factor (BF_01_) of the null for the effect of group for men was 5.18 for overall retest performance and was 5.06 for the proportion of errors corrected. These results suggest that both of the null hypotheses were over 5 times stronger than the alternatives (Jarosz and Wiley, 2014). Therefore, there was substantial support that the absence of an effect for men was not merely due to a lack of power.

#### Error Correction: Rewarded vs. Non-rewarded Errors

Given that total error correction includes both the correction of rewarded and non-rewarded errors, we conducted an additional analysis to determine whether the effects observed in the overall error correction analysis were primarily driven by errors that were rewarded, or extended equally to those that were not. Because the total number of non-rewarded errors included responses that were eligible for reward (∼58% of non-rewarded errors) as well as lower-quality ineligible responses (∼42% of non-rewarded errors), we conducted two analyses. In the first, we directly compared error correction rates for rewarded and non-rewarded trials as a function of group and gender, and thus, opted to only include reward-eligible non-rewarded responses. Reward-eligible non-rewarded trials are more directly comparable to the rewarded trials on dimensions of answer quality and domain familiarity (i.e., greater domain familiarity for reward eligible trials). In the second analysis, we conducted analyses on rewarded and non-rewarded trials separately, which allowed us to combine reward-eligible and reward-ineligible trials in our analysis of non-rewarded trials, given that both trial types represent an opportunity for error correction. This separate analysis also minimizes any concerns regarding the unequal number of rewarded and non-rewarded trials.

When directly comparing rewarded and reward-eligible non-rewarded trials, we found no significant main effects or interactions (all *F*s < 1.7, *p*s > 0.2, η*p*^2^s < 0.03). Thus, not only did this analysis show that our reward manipulation had no differential trial-level effects across rewarded and non-rewarded trials, but the necessary exclusion of reward-ineligible trials appeared to reduce the magnitude of overall benefits of the Effort group on women’s error correction. Indeed, when separately analyzing rewarded trials and all non-rewarded trials (i.e., allowing inclusion of both reward-eligible and reward-ineligible trials), we now found that for non-rewarded trials, although no main effects were observed (all *F*s < 0.4, *p*s > 0.75, η*p*^2^s < 0.01), a trend toward an interaction between group and gender emerged, *F*(3, 132) = 291, *p* < 0.096, η*p*^2^ = 0.047. *Post hoc* comparisons of these non-rewarded errors demonstrated a pattern identical to that seen for overall errors (see section “Overall Performance”). Specifically, women in the Effort group corrected significantly more non-rewarded errors (*M* = 0.81, *SD* = 0.06) than both men in the Effort group (*M* = 0.74, *SD* = 0.13, *p* = 0.039) and women in the Control group (*M* = 0.73, *SD* = 0.11, *p* = 0.027). A similar pattern was found when analyzing rewarded trials alone. Although there were no significant main effects (all *F*s < 1.2, *p*s > 0.31, η*p*^2^s < 0.03), there was a marginally significant interaction between group and gender, *F*(3, 132) = 2.35, *p* = 0.076, η*p*^2^ = 0.051. *Post hoc* exploration of this interaction confirmed that women in the Effort group (*M* = 0.82, *SD* = 0.09) corrected significantly more rewarded errors than men in the same group (*M* = 0.72, *SD* = 0.12, *p* = 0.011), although the difference between women in the Effort and Control groups did not reach significance.

Taken together, these analyses provide little evidence that the presentation of a reward-related stimulus after the feedback has a circumscribed trial-level effect on encoding of that particular learning opportunity. Rather to the extent that women benefitted when the task was framed as rewarding effortful responses, that benefit appeared to extend even to trials where no reward was presented, and regardless of whether the answer on that trial was reward-eligible or not.

#### Error Correction: High- vs. Low-Confidence Errors

We also predicted that error remediation might vary depending on the participant’s initial confidence in their erroneous response (i.e., greater for higher confidence errors; Butterfield and Metcalfe, 2001). Because participants may calibrate their confidence differently along the scale, we defined higher and lower confidence errors for each participant individually, based on a median split of their confidence ratings for incorrect responses^3^ (Median confidence value: *Mode* = 4, *M* = 4.1, *SD* = 0.88; see also Mangels et al., 2006). Median confidence values did not systematically differ across groups or gender (all *F*s < 1, *p*s > 0.33, η*p*^2^s < 0.007), and indeed overall metacognitive sensitivity was also similar across groups at both the initial test and retest (see Supplementary Section “S7. Metacognitive Sensitivity”). Having assured that groups were matched on these overall confidence factors, we then compared error correction rates with confidence (high, low) as a within-subjects factor, alongside group and gender as between-subjects factors.^4^

**FIGURE 3:**
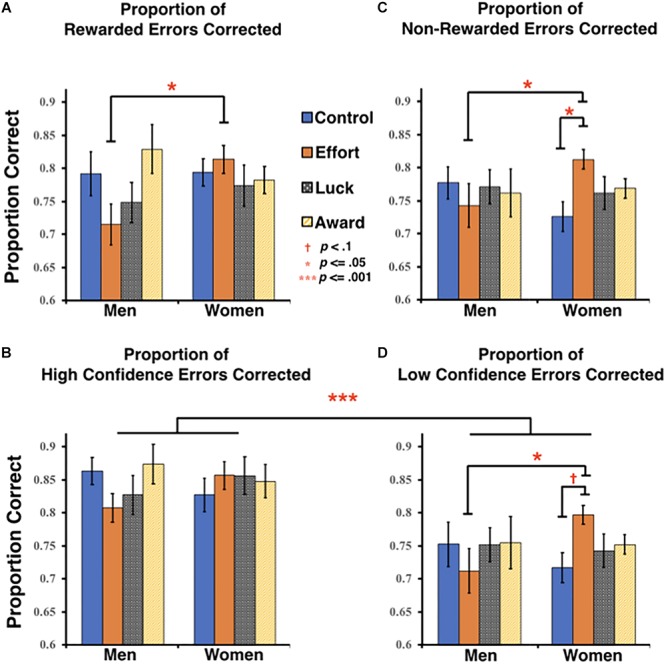
Trial-level analyses of error correction. Proportion of errors corrected on trials in which **(A)** a reward was received and **(B)** no reward was received (non-rewarded errors include both reward-eligible and ineligible trials). Proportion of errors corrected for trials endorsed either with **(C)** high confidence or **(D)** low confidence. Error bars represent the standard error of the mean (SEM).

As shown in the lower panels of Figure 3, participants corrected significantly more high confidence errors than low confidence errors overall, *F*(1, 132) = 82.02, *p* < 0.001, η*p*^2^ = 0.383. This analysis also included the previously reported marginally significant overall group x gender interaction on error correction, *F*(3, 132) = 2.13, *p* = 0.099, η*p*^2^ = 0.046 (see also section “Overall performance”), but did not find any other significant or marginal main effects or interactions (all *F*s < 0.58, *p*s > 0.63, η*p*^2^s < 0.67). Nonetheless, given that there may be somewhat of a ceiling effect with respect to the correction of high confidence items, and we had an *a priori* prediction that the benefits conferred by reward framing might be greater for the low confidence errors, we explored the effects of gender and group at each confidence level separately. A univariate ANOVA conducted on high confidence errors alone found no significant or marginal effects of group and/or gender (*F*s < 1.15, *p*s > 0.33, η*p*^2^s < 0.03; Figure 3C). However, for low confidence errors, we once again observed the marginal interaction between group and gender, *F*(3, 132) = 2.18, *p* = 0.093, η*p*^2^ = 0.047 (Figure 3D). Further *post hoc* exploration of this effect indicated that women in the Effort group corrected significantly more low confidence errors (*M* = 0.80, *SD* = 0.07) than men in the Effort group (*M* = 0.71, *SD* = 0.13, *p* = 0.016) and marginally more errors than women in the Control group (*M* = 0.72, *SD* = 0.11, *p* = 0.07). No other significant or marginal effects of group and/or gender were found for the proportion of low confidence errors corrected (*F*s < 0.3, *p*s > 0.86, η*p*^2^s < 0.01).

### Post-block/Post-test Questions

Table 2 provides descriptive statistics for all post-block and post-test questions. Data was missing from one subject in the Effort group for all post-block questions and from 9 participants (3 from the Control group and 2 from each of the other groups) for each of the post-test questions. The data loss was due to scripting errors with our testing and questionnaire programs, and not due to the participants declining to respond. The analyses below include the remaining participants (139 post-block; 131 post-test).

**TABLE 2:** Post-Block (first test) and Post-Retest Questions.

	**Men**				**Women**				
**Post-Block**	**Control**	**Effort**	**Luck**	**Award**	**Control**	**Effort**	**Luck**	**Award**	**Post Hoc Comparisons**
Ability to Concentrate	4.9 (0.9)	5.7 (1.0)	5.2 (1.7)	4.3(1.9)	4.9(1.3)	6.0(1.0)	5.4 (1.2)	5.1 (1.2)	E > C^∗∗^, E > A^∗∗^
Motivation to Perform Well	5.8 (1.1)	6.2 (0.7)	5.2 (1.8)	5.3 (2.1)	5.5 (1.1)	6.2 (0.7)	5.9 (0.8)	5.7 (1.1)	*n.s.*
Question Difficulty	4.6 (0.5)	4.7 (0.8)	4.5 (1.3)	4.1 (1.5)	4.7 (0.7)	4.6 (0.7)	4.6 (0.7)	4.8 (0.8)	*n.s.*
Performance Level	4.0 (0.7)	4.3 (0.8)	3.6 (1.1)	3.4 (1.5)	3.5 (0.9)	4.1 (0.8)	3.8 (0.7)	3.5 (0.8)	E > A^*^
Positive Affect when Correct	5.8 (0.9)	5.8 (0.7)	5.4 (1.7)	5.1 (2.0)	5.8 (0.8)	6.1 (0.8)	5.6 (1.0)	5.8 (0.9)	*n.s.*
Negative Affect when Wrong	2.8 (1.0)	3.2 (1.1)	2.8 (1.0)	2.5 (1.2)	3.0 (0.9)	3.0 (0.6)	3.2 (0.7)	3.0 (0.8)	*n.s.*
Positive Affect when Rewarded	5.3 (0.9)	6.0 (0.9)	5.6 (0.8)	5.0 (1.4)	5.2 (1.1)	5.9 (0.8)	5.4 (0.9)	5.5 (1.0)	E > C^∗∗^, E > A^*^
Negative Affect when Not Rewarded	3.7 (0.6)	3.5 (0.9)	3.5 (0.6)	3.3 (0.8)	3.5 (0.8)	3.1 (0.8)	3.7 (0.5)	3.4 (0.7)	*n.s.*
**Post-Retest**									
Trying Hard (Effort)	5.7 (2.3)	6.6 (2.6)	6.9 (2.0)	6.3 (2.2)	6.0 (1.7)	7.3 (2.1)	7.0 (1.4)	6.5 (2.1)	E > C^*^
Question Difficulty	2.0 (1.2)	2.3 (1.3)	2.4 (0.9)	2.0 (1.2)	2.6 (1.6)	2.4 (1.5)	3.3 (1.6)	2.4 (1.0)	*n.s.*
Performance Relative to Others	6.5 (1.2)	6.9 (1.2)	7.5 (1.3)	7.3 (1.3)	6.8 (1.3)	6.9 (1.4)	7.2 (1.1)	6.6 (1.6)	*n.s.*
Expected Retest	2.5 (2.4)	1.6 (1.4)	1.9 (1.7)	2.6 (2.7)	2.3 (2.0)	1.8 (1.9)	1.8 (1.5)	1.8 (1.9)	*n.s.*
Deliberately Studied Between Tests	1.5 (0.7)	1.5 (0.7)	1.7 (1.6)	2.1 (2.2)	1.4 (1.0)	2.1 (2.1)	1.6 (1.2)	2.0 (1.9)	*n.s.*

Mean ratings are shown with standard deviations in parentheses. Post-Block ratings were collected using a 7-point Likert scale. Although we tailored the exact wording of each scale to the concept being questioned, for all items except the affect questions, the low end of the scale (1) corresponded to the lowest level of that concept (i.e., “not able to concentrate at all,” “not at all motivated,” “questions not at all difficult/very easy,” “performed very poorly”), whereas the high end of the scale (7) corresponded to the highest level of that concept (i.e., “concentrated very well,” “highly motivated,” “questions very difficult,” “performance was excellent”), and the middle value (4) corresponded to a “moderate” level of each concept. For the affect-oriented questions, however, the low end of the scale always corresponded to negative affect (i.e., “very upset”) and the high end of the scale to positive affect (i.e., “very happy”), with the middle value representing “indifference,” regardless of whether the question asked about positive or negative affect. All Post-Retest ratings were collected using the same 9-point Likert scale (1 = “not at all”, 5 = “somewhat”, 9 = “extremely”), with the exception of the question about performance (relative to peers), which was scaled from 1 = “much worse” to 9 = “much better,” with 4 = “about the same”). All significant values are Bonferroni corrected for multiple comparisons. Significant post hoc tests are indicated with (^*^) where ^*^ = p < 0.05 and ^∗∗^ = p < 0.01. Multivariate tests and post hoc tests for Post-Block and Post-Retest Questions were conducted on rank transformed data.Non-transformed means and standard deviations are reported in the table. For Post Hoc Comparisons, groups are indicated by first initial (C = Control, E = Effort, L = Luck, A = Award).

#### Post-block Subjective Experience

Across all post-block questions, the MANOVA revealed a marginal effect of group, Pillai’s Trace = 0.254, *F*(24, 360) = 1.46, *p* = 0.078, η*p*^2^ = 0.085, but no effect of gender or group by gender interaction (*F*s < 1.6, *p*s > 0.15, η*p*^2^ < 0.09). When we explored the marginal effect of group further, univariate tests revealed significant group differences in concentration *F*(3,131) = 5.78, *p* = 0.001, η*p*^2^ = 0.117, perceived performance, *F*(3,131) = 2.73, *p* = 0.046, η*p*^2^ = 0.059, and positive affect following rewards *F*(3,131) = 4.73, *p* = 0.006, η*p*^2^ = 0.091. Following up these findings with *post hoc* comparisons, we found that, regardless of gender, participants in the Effort group reported significantly higher levels of concentration, perceived performance, and positive affect upon reward receipt compared to the Award group, and higher levels of concentration and positive affect than the Control group (see Table 2). No other comparisons were significant.

#### Post-test Subjective Experience

Similar to the post-block questions, a MANOVA of the questions asked after the retest revealed a significant effect of group, Pillai’s Trace = 0.2, *F*(15, 363) = 1.72, *p* = 0.045, η*p*^2^ = 0.067, but no main effect of gender or group by gender interaction (All, Pillai’s Trace <0.05, *F*s < 1.0, *p*s > 0.5, η*p*^2^s < 0.04). Univariate tests indicated that significant group differences were only found for subjective reports of effort, *F*(3, 123) = 2.81, *p* = 0.042, η*p*^2^ = 0.064. *Post hoc* comparisons further indicated participants in the Effort group reported giving significantly higher levels of effort on the retest than participants in the Control group (see Table 2). No other mean differences between groups were significant.

## Discussion

The present study was interested in testing a fundamental question relevant to the intersection of education and cognitive psychology: can explicit rewards for meaningful attempts at knowledge retrieval that nonetheless lead to wrong answers, ultimately facilitate learning of the right answer? There is substantial research showing that active testing yields better learning outcomes compared to passive review (Hogan and Kintsch, 1971; McDaniel et al., 2007), yet we know of no previous research that has specifically examined the effects of explicitly rewarding a student for retrieval attempts based on their answer quality, which we define here as evidence of retrieval “effort.” Likewise, although a number of recent studies have examined the effects of performance-contingent rewards on encoding in declarative memory (e.g., Murty and Adcock, 2013; Miendlarzewska et al., 2016), none as of yet have explored the circumstances under which such effort-contingent rewards might facilitate error remediation. Drawing on Cognitive Evaluation Theory (CET; Deci, 1975), we predicted that rewards tied to a student’s self-determined attempts to engage meaningfully with the initial retrieval phase of a general knowledge task (i.e., Effort group) would be particularly beneficial in facilitating incidental encoding of correct answer feedback, as demonstrated on a later surprise retest for this information. To tease apart the cognitive components of any observed benefit of these effort-contingent rewards, we compared the Effort group to a Luck group, where participants were told that their potential for reward was tied to some degree of choice (internal control), but not to answer competence, an Award group where rewards were given randomly (related to neither internal control, nor competence), and a Control group designed to control for the basic novelty/perceptual salience of the reward stimuli.

We found some support for our predictions, but this support was limited to women, and statistically strongest when considering retest performance for all items, rather than the correction of initial errors. Specifically, women in the Effort group significantly outperformed women in the Control group in terms of overall retest performance. They also outperformed men in the Effort group, who showed no evidence of benefit from any type of reward as a group. Similar patterns were found when focusing our analysis only on correction of initial errors. However, although the difference between women in the Effort and Control groups was on average ∼6%, which equates to a difference of about 4–5 items, this difference was only statistically marginal likely due to the greater variability in error correction performance than overall performance. In both analyses, women’s retest performance in the Luck and Award groups was not statistically distinguishable from the Effort or Control groups, suggesting that neither internal control alone nor reward in isolation accounted wholly for the influence of the effort-contingent framing on women’s performance. Rather, it appears that the combination of these factors was necessary to achieve the full benefits observed for this group. However, we cannot rule out that there was some additional factor in this group, not controlled for by the comparison groups, which also contributed to its learning benefits.

Additionally, in considering why the effects of the Effort group on overall retest performance might have been more statistically robust, compared to error correction alone, we note that overall retest performance includes both the correction of first-test errors as well as maintenance of initially accurate responses (∼50% of first-test trials), some of which the participant likely knew confidently and others for which they may have been guessing or were less certain. For women in the Effort group, the reward framing may have both facilitated the learning from feedback on initially incorrect responses, as well as reinforced the encoding of correct answers. Although lower confidence correct responses may have been particularly sensitive to reward reinforcement, they are typically relatively few in number, which precludes systematic analysis. However, a larger range of confidence values for initial errors permitted analysis of the relationship between reward framing, gender and initial response confidence in relation to error remediation specifically, which we discuss below.

An exploratory analysis examining the effects of reward framing and gender on error remediation for low- and high- confidence errors separately suggested that any benefits of the Effort group experienced by women were primarily driven by the enhanced remediation of low-confidence errors. Although high-confidence errors were corrected overwhelmingly more often than low-confidence errors regardless of gender, replicating the hypercorrection effect (e.g., Butterfield and Metcalfe, 2001, 2006; Butterfield and Mangels, 2003), error correction of these items did not appear to be modulated further by reward framing. The relative rarity of high-confidence errors already up-regulates attention to learning more than their low-confidence counterparts (e.g., Butterfield and Mangels, 2003; Butterfield and Metcalfe, 2006), and correct answer feedback is more likely to benefit from processing fluency associated with the greater domain familiarity associated with these types of errors (Unkelbach and Greifeneder, 2013). Thus, it was not unexpected that any influence of reward on attention would have relatively little additional effect. However, for feedback following low-confidence errors, which have neither the intrinsic qualities of surprise nor semantic fluency, an effort-contingent framing resulted in significantly greater error correction for women compared to men, and marginally greater error correction when comparing women in the Effort and Control groups directly.

These results mirror the overall error correction findings discussed above (i.e., when all items were considered, regardless of confidence) and is potentially consistent with previous studies (Murayama and Kuhbandner, 2011) where extrinsic rewards appear to have the greatest effect on learning when the information to-be-learned is of low intrinsic interest. Current models of reward modulation in declarative memory suggest that positive rewards particularly benefit associative encoding (for review see Murty and Adcock, 2013; Miendlarzewska et al., 2016; Chiew and Adcock, 2019). Low-confidence error correction could be construed as placing greater demands on episodic encoding of new question-answer associations compared to high-confidence errors whose greater domain familiarity could capitalize more on activation of pre-existing semantic relationships.

Additionally, to inform our understanding of the potential mechanisms underlying any observed framing effects, we examined the effects of reward at the trial-level by comparing the proportion of rewarded and non-rewarded errors corrected at retest. Based on research supporting the dopaminergic memory consolidation process (e.g., Murayama and Kuhbandner, 2011; Shigemune et al., 2013; Murayama and Kitagami, 2014), we had anticipated that rewarded errors might be corrected more often than non-rewarded errors. However, the promise of extrinsic rewards may also increase general motivation toward the task as a whole conferring an overall benefit of reward framing as opposed to benefitting specific trial types (Locke and Braver, 2008; but see Kostandyan et al., 2019). In support of a general motivation effect, we found no evidence of differentially greater error correction for rewarded compared to non-rewarded trials either overall or as a function of framing or gender. Indeed, further exploration of framing and gender effects within rewarded and non-rewarded trials separately found that effort-contingent framing benefitted the error correction of women significantly more than men for both rewarded and non-rewarded trials (at least when both reward eligible and ineligible trials were considered together). If anything, the effect of framing rewards as effort-contingent was more statistically robust for women on these non-rewarded trials, given that it was only for these trials that the Effort and Control groups differed significantly from each other. This finding is also broadly consistent with our previous findings with respect to low-confidence errors. Insofar as our effort manipulation was successful in motivating overall task engagement, this may have facilitated greater attention to corrective feedback for those items with little intrinsic salience, including non-rewarded trials and trials with low initial confidence.

### Limitations and Future Directions

It was somewhat surprising that effort-contingent reward framing conferred a retest performance advantage for women only, given that both women and men in this group reported greater task concentration in general, and more positive feelings about rewards in particular, than participants in either the Award or Control groups. Both women and men in the Effort group also self-reported giving greater effort on the retest compared to the Control group. These self-report measures would suggest that both men and women in the Effort group felt more engaged with the task than participants in the other groups, yet only women demonstrated the positive effects of this engagement in terms of subsequent memory. Although interpretation of these gender differences should be made with caution given the differences in sample sizes, our analyses indicated that it is unlikely that increasing this sample would yield effects for men that simply mirrored those of women. Moreover, across all of our analyses the mean scores for men in the Effort group were lower than men in any other framing group whereas the scores for women in the Effort group were higher than women in any other framing group.

One possibility is that these gender-specific performance differences may be a result of the general “goodness of fit” between our Effort group and the intrinsic academic goals more typically ascribed to women (Vallerand, 1989; Vallerand et al., 1992). Women also tend to demonstrate greater verbal and language arts self-concepts (Skaalvik and Skaalvik, 2004), and perform better on verbal episodic memory tasks (Herlitz et al., 1997; Lewin et al., 2001; Herlitz and Rehnman, 2008) including within similar paradigms to the present study (Whiteman and Mangels, 2016; Mangels et al., 2018). Together, these factors may have made women particularly well-suited to thrive in our Effort group, in which intrinsically motivating factors of internal control and competency were highlighted within a challenging verbal episodic memory task. Replication of these gender interactions and extension across a wider range of tasks could be particularly useful for educators aimed at finding methods to maximize learning and engagement in students.

To the extent that rewards influenced learning in this study, it appeared to be through general motivational mechanisms rather than trial-level influences. A number of design factors may have limited the trial-level effects of the reward manipulation. Principally, we opted to use an indirect, deferred, and cumulative reward (i.e., earning increased chances at a later raffle for money), rather than an immediate, tangible reward for each item (e.g., money for each effortful response). While our approach may have greater practicality in the classroom setting, it may have limited item-level reward salience and instead, focused attention to the overall task level. The timing of reward presentation *after* the presentation of the to-be-remembered stimulus (i.e., the correct answer), as well as the incidental nature of the learning task, may also have reduced the ability of the reward to “tag” encoding of a specific item. Although some studies have found that rewards may enhance learning for information merely presented within the same context, even without necessarily motivating attention directly toward the to-be-learned information (i.e., a “penumbra” effect; Murty and Adcock, 2013; Murayama and Kitagami, 2014), these effects may not be as strong as when the reward is contingent on learning or presented concurrently with the to-be-remembered stimulus. Future studies could examine the relative influence of reward timing by cueing the reward for effort either before, concurrently with, or immediately following the presentation of the correct answer to determine the context under which item-level responses might emerge.

We also note that the overall effects of the Effort group were not as robust as expected. Even though women’s error remediation was generally greater in the Effort group compared to the Control group, in some cases that difference was only marginal, and it did not differ statistically from either the Luck or Award groups. This may be due in part to the subtlety in instruction variation across the four groups. In an effort to control for overall motivation, instructions in all four groups indicated that presentation of the rare, task-relevant stimulus (i.e., the reward in Effort, Luck and Award groups; the to-be-counted stimulus for the Control group) would be contingent on overall performance in the task. Specifically, this stimulus would be more likely to appear the better the participant did in the task overall. Inclusion of a neutral group where either no post-feedback stimuli were provided, and/or no general instruction to perform well was given, might provide a better contrast in future studies.

Finally, to the extent that beneficial effects of rewards for effort were observed in women, it is not clear how long lasting these effects might be. Our retest occurred after a 24–48 h period, but it is unknown whether the influence of this reward would persist over the longer delays most relevant to classroom learning. It would also be interesting to consider whether rewards for effort might be particularly beneficial when learning requires sustained attention. At 160 questions, our task was fairly lengthy, and likely placed reasonable demands on effortful persistence, but future studies might consider more formally examining the relationship between task length, reward schedule and learning outcomes. Future work might also consider how persistent use of an explicit effort-contingent reward might either make a mastery approach habitual and intrinsic or, in contrast, could actually serve to undermine intrinsic motivation to exert effort toward difficult tasks in the same way that extrinsic motivators have been shown to reduce other types of intrinsically motivated behaviors (Deci, 1975; Deci et al., 1999, 2001).

## Conclusion

To our knowledge, this is the first study to consider how extrinsic rewards that are effort-contingent, rather than performance-contingent, can benefit long-term memory for declarative information. As such, it addresses the intersection of achievement motivation, reward-based learning systems, and test-enhanced (i.e., feedback-based) learning of declarative knowledge. Furthermore, it goes beyond treating extrinsic rewards as a unidimensional motivational stimulus, and rather, leverages the predictions of Cognitive Evaluation Theory to consider the joint roles of competence and self-determination in relationship to reward significance (Deci, 1975).

While our behavioral results were not as strong as expected with respect to group-level differences, our findings suggest there may be considerable opportunity for future research to explore the nuanced relationships between reward, intrinsic motivation, incidental learning and gender. This line of research has obvious implications within academic settings in which discovering the best ways to motivate students to expend effort toward learning is a key objective. CET and related theories have been quite influential over the past couple of decades; especially within academics (Grolnick and Ryan, 1987; Reeve, 2012; Saeed and Zyngier, 2012). However, much remains to be understood with regards to how reward contexts shape the learning environment, influence motivation over the long-term, and affect learning and declarative memory.

## Ethics Statement

The study was carried out at the University of Denver in accordance with the recommendations from the University of Denver Institutional Review Board (DU IRB) with written informed consent from all participants. All participants gave written informed consent in accordance with the Declaration of Helsinki. The protocol was approved by the University of Denver Institutional Review Board (DU IRB).

## Author Contributions

DA and JM contributed to the conception and design of the study, analyzed the data, and wrote the manuscript. DA collected the study data. KM contributed to study design, interpretation related to the analysis, and editing of the manuscript.

## Conflict of Interest Statement

The authors declare that the research was conducted in the absence of any commercial or financial relationships that could be construed as a potential conflict of interest.

## Footnotes

1 www.mangelslab.org/bknorms

2 www.mangelslab.org/bknorms

3 The median confidence score was included either in the higher confidence or lower confidence bin depending on which gave better balance with respect to the number of higher and lower confidence trials in the analysis.

4 Because higher confidence errors were relatively rare, even when defined by a median split procedure, we did not have sufficient power to further separate high and low confidence errors into rewarded and non-rewarded subcategories.

## References

[B1] Adcock R. Thangavel A. Whitfield-Gabrieli S. Knutson B. Gabrieli J. (2006). Reward-motivated learning: mesolimbic activation precedes memory formation. *Neuron* 50 507–517. 10.1016/j.neuron.2006.03.036 16675403

[B2] Angrist J. Oreopoulos P. Williams T. (2014). When opportunity knocks, who answers?: new evidence on college achievement awards. *J. Hum. Resour.* 49 572–610. 10.1353/jhr.2014.0019

[B3] Bunzeck N. Dayan P. Dolan R. Duzel E. (2010). A common mechanism for adaptive scaling of reward and novelty. *Hum. Brain Mapp.* 31 1380–1394. 10.1002/hbm.20939 20091793 PMC3173863

[B4] Butterfield B. Mangels J. A. (2003). Neural correlates of error detection and correction in a semantic retrieval task. *Cogn. Brain Res.* 17 793–817. 10.1016/S0926-6410(03)00203-9 14561464

[B5] Butterfield B. Metcalfe J. (2001). Errors committed with high confidence are hypercorrected. *J. Exp. Psychol. Learn. Mem. Cogn.* 27 1491–1494. 10.1037/0278-7393.27.6.1491 11713883

[B6] Butterfield B. Metcalfe J. (2006). The correction of errors committed with high confidence. *Metacogn. Learn.* 1 69–84. 10.1007/s11409-006-6894-z 11713883

[B7] Chiew K. S. Adcock R. A. (2019). “Motivated memory: integrating cognitive and affective neuroscience,” in *Cambridge Handbook of Motivation and Learning*, eds Ann Renninger K. Hidi Suzanne D. (Cambridge: Cambridge University Press).

[B8] Chiew K. S. Braver T. S. (2013). Temporal dynamics of motivation-cognitive control interactions revealed by high-resolution pupillometry. *Front. Psychol.* 4:15. 10.3389/fpsyg.2013.00015 23372557 PMC3557699

[B9] Chiew K. S. Braver T. S. (2016). Reward favors the prepared: incentive and task-informative cues interact to enhance attentional control. *J. Exp. Psychol. Hum. Percept. Perform.* 42 52–66. 10.1037/xhp0000129 26322689 PMC4688088

[B10] Cokley K. O. Bernard N. Cunningham D. Motoike J. (2001). A psychometric investigation of the academic motivation scale using a United States sample. *Meas. Eval. Couns. Dev.* 34 109–119. 10.1097/JPA.0000000000000012 25715010

[B11] Deci E. L. (ed.). (1975). “Cognitive evaluation theory: effects of extrinsic rewards on intrinsic motivation,” in *Intrinsic Motivation*, (Boston, MA: Springer), 129–159. 10.1007/978-1-4613-4446-9_5

[B12] Deci E. L. Koestner R. Ryan R. M. (2001). Extrinsic rewards and intrinsic motivation in education: reconsidered once again. *Rev. Educ. Res.* 71 1–28. 10.3102/00346543071001001

[B13] Deci E. L. E. Koestner R. R. Ryan R. M. R. (1999). A meta-analytic review of experiments examining the effects of extrinsic rewards on intrinsic motivation. *Psychol. Bull.* 125 627–700. 10.1037/0033-2909.125.6.627 10589297

[B14] Floresco S. B. West A. R. Ash B. Moore H. Grace A. A. (2003). Afferent modulation of dopamine neuron firing differentially regulates tonic and phasic dopamine transmission. *Nat. Neurosci.* 6 968–973. 10.1038/nn1103 12897785

[B15] Glimcher P. W. (2011). Understanding dopamine and reinforcement learning: the dopamine reward prediction error hypothesis. *Proc. Natl. Acad. Sci. U.S.A.* 108(Suppl._3), 15647–15654. 10.1073/pnas.1014269108 21389268 PMC3176615

[B16] Grolnick W. S. Ryan R. M. (1987). Autonomy in children’s learning: an experimental and individual difference investigation. *J. Pers. Soc. Psychol.* 52 890–898. 10.1037//0022-3514.52.5.890 3585701

[B17] Gruber M. J. Gelman B. D. Ranganath C. (2014). States of curiosity modulate hippocampus-dependent learning via the dopaminergic circuit. *Neuron* 84 486–496. 10.1016/j.neuron.2014.08.060 25284006 PMC4252494

[B18] Henderson V. L. Dweck C. S. (1990). *Motivation and Achievement.* Cambridge, MA: Harvard University Press.

[B19] Herlitz A. Nilsson L.-G. Bäckman L. (1997). Gender differences in episodic memory. *Mem. Cogn.* 25 801–811. 10.3758/BF03211324 9421566

[B20] Herlitz A. Rehnman J. (2008). Sex differences in episodic memory. *Curr. Dir. Psychol. Sci.* 17 52–56. 10.1111/j.1467-8721.2008.00547.x

[B21] Hogan R. M. Kintsch W. (1971). Differential effects of study and test trials on long-term recognition and recall. *J. Verbal Learn. Verbal Behav.* 10 562–567. 10.1016/S0022-5371(71)80029-4

[B22] Horvitz J. (2000). Mesolimbocortical and nigrostriatal dopamine responses to salient non-reward events. *Neuroscience* 96 651–656. 10.1016/S0306-4522(00)00019-1 10727783

[B23] Huang C. (2012). Gender differences in academic self-efficacy: a meta-analysis. *Eur. J. Psychol. Educ.* 28 1–35. 10.1007/s10212-011-0097-y 29154576

[B24] Jarosz A. F. Wiley J. (2014). What are the odds? A practical guide to computing and reporting bayes factors. *J. Probl. Solving* 7 1–8. 10.7771/1932-6246.1167

[B25] JASP Team (2018). *JASP (Version 0.9.2) [Computer Software].*

[B26] Kang M. Hsu M. Krajbich I. Lowenstein G. McClure S. Wang J. (2009). The wick in the candle of learning: epistemic curiosity activates reward circuitry and enhances memory. *Psychol. Sci.* 20 963–973. 10.1111/j.1467-9280.2009.02402.x 19619181

[B27] Kluger A. N. DeNisi A. (1998). Feedback interventions: toward the understanding of a double-edged sword. *Curr. Dir. Psychol. Sci.* 7 67–72. 10.1111/1467-8721.ep10772989

[B28] Knutson B. Westdorp A. Kaiser E. Hommer D. (2000). FMRI visualization of brain activity during a monetary incentive delay task. *Neuroimage* 12 20–27. 10.1006/nimg.2000.0593 10875899

[B29] Kostandyan M. Bombeke K. Carsten T. Krebs R. M. Notebaert W. Boehler C. N. (2019). Differential effects of sustained and transient effort triggered by reward – A combined EEG and pupillometry study. *Neuropsychologia* 123 116–130. 10.1016/j.neuropsychologia.2018.04.032 29709582

[B30] Krebs R. M. Woldorff M. G. (2017). “Cognitive control and reward,” in *The Wiley Handbook of Cognitive Control*, ed. Egner T. (Hoboken, NJ: John Wiley & Sons), 422–439. 10.1002/9781118920497.ch24

[B31] Lepper M. R. Greene D. Nisbett R. E. (1973). Undermining children’s intrinsic interest with extrinsic reward: a test of the “overjustification” hypothesis. *J. Pers. Soc. Psychol.* 28 129–137. 10.1037/h0035519

[B32] Levitt S. D. List J. A. Sadoff S. (2016a). *The Effect of Performance-Based Incentives on Educational Achievement: Evidence from a Randomized Experiment.* NBER Working Paper No. 22107.

[B33] Levitt S. D. List J. A. Neckermann S. Sadoff S. (2016b). The behavioralist goes to school: leveraging behavioral economics to improve educational performance. *Am. Econ. J. Econ. Policy* 8 183–219. 10.1257/pol.20130358

[B34] Lewin C. Wolgers G. Herlitz A. (2001). Sex differences favoring women in verbal but not in visuospatial episodic memory. *Neuropsychology* 15 165–173. 10.1037/0894-4105.15.2.165 11324860

[B35] Lisman J. Grace A. A. Duzel E. (2011). A neoHebbian framework for episodic memory; role of dopamine-dependent late LTP. *Trends Neurosci.* 34 536–547. 10.1016/j.tins.2011.07.006 21851992 PMC3183413

[B36] Lisman J. E. Grace A. (2005). The hippocampal-VTA loop: controlling the entry of information into long-term memory. *Neuron* 46 703–713. 10.1016/j.neuron.2005.05.002 15924857

[B37] Locke H. S. Braver T. S. (2008). Motivational influences on cognitive control: behavior, brain activation, and individual differences. *Cogn. Affect. Behav. Neurosci.* 8 99–112. 10.3758/CABN.8.1.99 18405050

[B38] Mangels J. A. Butterfield B. Lamb J. Good C. Dweck C. S. (2006). Why do beliefs about intelligence influence learning success? A social cognitive neuroscience model. *Soc. Cogn. Affect. Neurosci.* 1 75–86. 10.1093/scan/nsl013 17392928 PMC1838571

[B39] Mangels J. A. Good C. Whiteman R. C. Maniscalco B. Dweck C. S. (2011). Emotion blocks the path to learning under stereotype threat. *Soc. Cogn. Affect. Neurosci.* 7 230–241. 10.1093/scan/nsq100 21252312 PMC3277368

[B40] Mangels J. A. Hoxha O. Lane S. P. Jarvis S. N. Downey G. (2018). Evidence that disrupted orienting to evaluative social feedback undermines error correction in rejection sensitive women. *Soc. Neurosci.* 13 451–470. 10.1080/17470919.2017.1358210 28724323 PMC6154388

[B41] Mangels J. A. Rodriguez S. Ochakovskaya Y. Guerra-Carrillo B. (2017). Achievement goal task framing and fit with personal goals modulate the neurocognitive response to corrective feedback. *AERA Open* 3:2332858417720875.

[B42] Manohar S. G. Finzi R. D. Drew D. Husain M. (2017). Distinct motivational effects of contingent and noncontingent rewards. *Psychol. Sci.* 28 1016–1026. 10.1177/0956797617693326 28488927 PMC5510684

[B43] Martin-Soelch C. Szczepanik J. Nugent A. Barhaghi K. Rallis D. Herscovitch P. (2011). Lateralization and gender differences in the dopaminergic response to unpredictable reward in the human ventral striatum. *Eur. J. Neurosci.* 33 1706–1715. 10.1111/j.1460-9568.2011.07642.x 21453423 PMC3086965

[B44] Mather M. Schoeke A. (2011). Positive outcomes enhance incidental learning for both younger and older adults. *Front. Neurosci.* 5:129. 10.3389/fnins.2011.00129 22125509 PMC3221314

[B45] McDaniel M. A. Anderson J. L. Derbish M. H. Morrisette N. (2007). Testing the testing effect in the classroom. *Eur. J. Cogn. Psychol.* 19 494–513. 10.1080/09541440701326154

[B46] Metcalfe J. (2017). Learning from errors. *Annu. Rev. Psychol.* 68 465–489. 10.1146/annurev-psych-010416-044022 27648988

[B47] Metcalfe J. Finn B. (2011). People’s hypercorrection of high-confidence errors: did they know it all along? *J. Exp. Psychol. Learn. Mem. Cogn.* 37 437–448. 10.1037/a0021962 21355668 PMC3079415

[B48] Miendlarzewska E. A. Bavelier D. Schwartz S. (2016). Influence of reward motivation on human declarative memory. *Neurosci. Biobehav. Rev.* 61 156–176. 10.1016/j.neubiorev.2015.11.015 26657967

[B49] Miserandino M. (1996). Children who do well in school: individual differences in perceived competence and autonomy in above-average children. *J. Educ. Psychol.* 88 203–214. 10.1037/0022-0663.88.2.203

[B50] Moser J. S. Schroder H. S. Heeter C. Moran T. P. Lee Y.-H. (2011). Mind your errors: evidence for a neural mechanism linking growth mind-set to adaptive posterror adjustments. *Psychol. Sci.* 22 1484–1489. 10.1177/0956797611419520 22042726

[B51] Mueller C. M. Dweck C. S. (1998). Praise for intelligence can undermine children’s motivation and performance. *J. Pers. Soc. Psychol.* 75 33–52. 10.1037/0022-3514.75.1.33 9686450

[B52] Murayama K. Kitagami S. (2014). Consolidation power of extrinsic rewards: reward cues enhance long-term memory for irrelevant past events. *J. Exp. Psychol. Gen.* 143 15–20. 10.1037/a0031992 23421444

[B53] Murayama K. Kuhbandner C. (2011). Money enhances memory consolidation–But only for boring material. *Cognition* 119 120–124. 10.1016/j.cognition.2011.01.001 21292249

[B54] Murty V. P. Adcock R. A. (2013). Enriched encoding: reward motivation organizes cortical networks for hippocampal detection of unexpected events. *Cereb. Cortex* 24 2160–2168. 10.1093/cercor/bht063 23529005 PMC4089383

[B55] Niv Y. Joel D. Dayan P. (2006). A normative perspective on motivation. *Trends Cogn. Sci.* 10 375–381. 10.1016/j.tics.2006.06.010 16843041

[B56] Ochakovskaya Y. (2018). *Manipulating Goal States and Brain States: Using EEG and HD-tDCS to Investigate Mechanisms Underlying the Influence of Achievement Goals on Declarative Memory.* New York, NY: CUNY Academic Works Available at: https://academicworks.cuny.edu/gc_etds/2830

[B57] Patil A. Murty V. P. Dunsmoor J. E. Phelps E. A. Davachi L. (2016). Reward retroactively enhances memory consolidation for related items. *Learn. Mem.* 24 65–69. 10.1101/lm.042978.116 27980078 PMC5159660

[B58] Redondo R. L. Morris R. G. M. (2011). Making memories last: the synaptic tagging and capture hypothesis. *Nat. Rev. Neurosci.* 12 17–30. 10.1038/nrn2963 21170072

[B59] Reeve J. (2012). “A self-determination theory perspective on student engagement,” in *Handbook of Research on Student Engagement*, eds Christenson S. L. Reschly A. L. Wylie C. (Boston, MA: Springer), 149–172. 10.1007/978-1-4614-2018-7_7

[B60] Ryan R. M. (1982). Control and information in the intrapersonal sphere: An extension of cognitive evaluation theory. *J. Pers. Soc. Psychol.* 43 450–461. 10.1037/0022-3514.43.3.450

[B61] Ryan R. M. Mims V. Koestner R. (1983). Relation of reward contingency and interpersonal context to intrinsic motivation: a review and test using cognitive evaluation theory. *J. Pers. Soc. Psychol.* 45 736–750. 10.1037/0022-3514.45.4.736

[B62] Saeed S. Zyngier D. (2012). How motivation influences student engagement: a qualitative case study. *J. Educ. Learn.* 1 252–267. 10.5539/jel.v1n2p252

[B63] Schultz W. (1998). Predictive reward signal of dopamine neurons. *J. Neurophysiol.* 80 1–27. 10.1152/jn.1998.80.1.1 9658025

[B64] Schultz W. (2001). Reward signaling by dopamine neurons. *Neuroscientist* 7 293–302. 10.1177/107385840100700406 11488395

[B65] Schunk D. (1985). Self-efficacy and classroom learning. *Psychol. Sch.* 22 208–223. 10.1002/1520-6807(198504)22:2<208::aid-pits2310220215>3.0.co;2-7

[B66] Schunk D. H. Pintrich P. R. Meece J. L. (2008). *Motivation in Education: Theory, Research, and Applications*, 3rd Edn London: Pearson.

[B67] Shigemune Y. Tsukiura T. Kambara T. Kawashima R. (2013). Remembering with gains and losses: effects of monetary reward and punishment on successful encoding activation of source memories. *Cereb. Cortex* 24 1319–1331. 10.1093/cercor/bhs415 23314939 PMC3977621

[B68] Shim S. Ryan A. (2005). Changes in self-efficacy, challenge avoidance, and intrinsic value in response to grades: the role of achievement goals. *J. Exp. Educ.* 73 333–349. 10.3200/JEXE.73.4.333-349

[B69] Sitzman D. M. Rhodes M. G. Tauber S. K. Liceralde V. R. T. (2015). The role of prior knowledge in error correction for younger and older adults. *Aging Neuropsychol. Cogn.* 22 502–516. 10.1080/13825585.2014.993302 25558782

[B70] Skaalvik S. Skaalvik E. M. (2004). Gender differences in math and verbal self-concept, performance expectations, and motivation. *Sex Roles* 50 241–252. 10.1023/B:SERS.0000015555.40976.e6 3226426

[B71] Spreckelmeyer K. N. Krach S. Kohls G. Rademacher L. Irmak A. Konrad K. (2009). Anticipation of monetary and social reward differently activates mesolimbic brain structures in men and women. *Soc. Cogn. Affect. Neurosci.* 4 158–165. 10.1093/scan/nsn051 19174537 PMC2686229

[B72] Stanek J. K. Dickerson K. C. Chiew K. S. Clement N. J. Adcock R. A. (2019). Expected reward value and reward uncertainty have temporally dissociable effects on memory formation. *J. Cogn. Neurosci.* 265, 1–12. 10.1162/jocn_a_01411 30990388 PMC7273969

[B73] Unkelbach C. Greifeneder R. (eds). (2013). *The Experience of Thinking: How the Fluency of Mental Processes Influences Cognition(and)Behaviour.* Hove: Psychology Press.

[B74] Vallerand R. J. (1989). Vers une méthodologie de validation trans-culturelle de questionnaires psychologiques: implications pour la recherche en langue française. *Can. Psychol.* 30, 662–680. 10.1037/h0079856 6850979

[B75] Vallerand R. J. Pelletier L. G. Blais M. R. Briere N. M. Senecal C. Vallieres E. F. (1992). The academic motivation scale: a measure of intrinsic, extrinsic, and amotivation in education. *Educ. Psychol. Meas.* 52 1003–1017. 10.1177/0013164492052004025

[B76] Wang S. H. Redondo R. L. Morris R. G. M. (2010). Relevance of synaptic tagging and capture to the persistence of long-term potentiation and everyday spatial memory. *Proc. Natl. Acad. Sci. U.S.A.* 107 19537–19542. 10.1073/pnas.1008638107 20962282 PMC2984182

[B77] Whiteman R. Mangels J. A. (2016). Rumination and rebound from failure as a function of gender and time on task. *Brain Sci.* 6:E7. 10.3390/brainsci6010007 26901231 PMC4810177

[B78] Wilgenbusch T. Merrell K. W. (1999). Gender differences in self-concept among children and adolescents: a meta-analysis of multidimensional studies. *Sch. Psychol. Q.* 14 101–120. 10.1037/h0089000

[B79] Wise S. L. Kong X. (2005). Response time effort: a new measure for examinee motivation in computer-based tests. *Appl. Meas. Educ.* 18 163–183. 10.1207/s15324818ame1802_2

[B80] Wittig M. Marks G. Jones G. (1981). Luck versus effort attributions. *Pers. Soc. Psychol. Bull.* 7 71–78. 10.1177/014616728171011

[B81] Wittmann B. Schott B. Guderian S. Frey J. Heinze H. Düzel E. (2005). Reward-related FMRI activation of dopaminergic midbrain is associated with enhanced hippocampus-dependent long-term memory formation. *Neuron* 45 459–467. 10.1016/j.neuron.2005.01.010 15694331

[B82] Wittmann B. C. Dolan R. J. Duzel E. (2011). Behavioral specifications of reward-associated long-term memory enhancement in humans. *Learn. Mem.* 18 296–300. 10.1101/lm.1996811 21502336 PMC3465832

[B83] Wolf L. F. Smith J. K. Birnbaum M. E. (1995). Consequences of performance, test motivation, and mentally taxing items. *Appl. Meas. Educ.* 8 341–351. 10.1207/s15324818ame0804_4

