# Supplementary material for: “A” for Effort: Rewarding Effortful Retrieval Attempts Improves Learning From General Knowledge Errors in Women
Source: Front Psychol. 2019 Jun 21;10:1179. doi: 10.3389/fpsyg.2019.01179 (PMC6598502; doi:10.3389/fpsyg.2019.01179)
Supplement: Supplementary file 1 [file Data_Sheet_1.pdf]

Supplementary Material

"A" For Effort: Incidental Learning from General Knowledge Errors is Enhanced When Women are Rewarded for Effortful Retrieval Attempts

Damon Abraham, Kateri McRae, Jennifer Mangels*

*** Correspondence:** Jennifer Mangels: jennifer.mangels@baruch.cuny.edu

S1. Questionnaires

Prior to the start of the first test, participants responded to a series of computerized validated motivation questionnaires including: the Work Preference Inventory (WPI; Amabile, Hill, Hennessey, & Tighe, 1994), which assesses intrinsic and extrinsic motivational orientations; the Revised Achievement Goals Questionnaire (AGQ; Elliot & Murayama, 2008), which measures mastery and performance academic achievement goals crossed with approach and avoidance motivations; the Emotion Regulation Questionnaire (ERQ; Gross & John, 2003), which measures the habitual use cognitive reappraisal and expressive suppression emotion regulation strategies; the Positive and Negative Affect Schedule (PANAS; Crawford & Henry, 2004), which measures both positive and negative affect; and the BIS/BAS scales (Carver & White, 1994), which are measures of behavioral inhibition and behavioral approach tendencies respectively. Questions pertaining to the BAS measure are divided across three subcomponents: 1) BAS Drive, which measures outward motivation (e.g., "I go out of my way to get things I want"), 2) BAS Reward Responsiveness, which measures how reward motivated the subject is (e.g., "When I get something I want, I feel excited and energized"), and 3) BAS Fun Seeking, which is a measure of approach tendencies towards exciting activities (e.g. "I crave excitement and new sensations"). At the conclusion of the first day's general knowledge task, subjects also completed the Intrinsic Motivation Inventory (IMI; Ryan, 1982), which measures the participant's intrinsic motivation for a specific task (e.g., the general knowledge task). The questions on the IMI are divided into four subcomponents: Enjoyment, Effort, Competence, and Tension.

We examined the questionnaires as possible moderating variables influencing performance on the primary task and ensured that these potentially influential individual characteristics were balanced across the four study groups. No significant main effects or interactions were found for any of the subscale measures of either the BIS/BAS, AGQ, ERQ, PANAS, or WPI pretest questionnaires or for the IMI posttest questionnaire. Scores on each subscale were consistent across the four study groups and there were no significant differences between genders (All Fs < 1.76, all ps > .10).

Multiple regression analysis was used to test whether the individual traits measured by the questionnaires significantly predicted participants' overall retest performance regardless of gender or group. The mean-centered subscale measures from each questionnaire were entered as separate predictors into the model (NB: only the primary subscales of Intrinsic and Extrinsic Motivation were entered for the WPI).

Table 1 compares the results of the regression analyses for two models. In the first model all subscale measures were included as predictors. The results of the regression indicated the predictors explained 17% of the variance (R^2 =.171, Adjusted R^2 = .045, F(18,119)= 1.36, p = .164). Although the model was non-significant, it was found that two variables significantly predicted retest performance. Higher endorsement of a performance approach achievement goal positively predicted

retest performance (β = .014, p = .038), whereas an extrinsic motivational orientation negatively predicted retest performance (β = -.04, p = .006).

In the second model, predictors were selectively eliminated via the backward stepwise regression procedure. The final stepwise model indicated that three predictors explained 14% of the variance (R^2 =.136, Adjusted R^2 = .116, $F(3,137)$= 7.01, p < .001). As in the first model, endorsement of a performance approach achievement goal significantly predicted retest performance (β = .014, p = .008) and an extrinsic motivational orientation remained a significant negative predictor of performance (β = -.04, p < .001). Likewise, a tendency to employ cognitive reappraisal as an emotion regulation strategy also significantly predicted retest performance (β = .009, p = .05), whereas this tendency was only a marginal predictor in the first model.

Table 1. Questionnaire Measures as Predictors of Overall Retest Performance. Significant tests indicated with (*) where * = p < .05, ** = p < .01, and *** = p < .001.

Variables	Model I		Model II	
	β	VIF	β	VIF
**PANAS Pos. Affect**	-0.001	1.5	-	-
**PANAS Neg. Affect**	0.000	1.3	-	-
**AGQ Mastery Approach**	-0.001	2.0	-	-
**AGQ Mastery Avoid**	0.005	1.6	-	-
**AGQ Performance Approach**	**0.014***	2.9	**0.298****	1.7
**AGQ Performance Avoid**	0.000	3.2	-	-
**BIS/BAS Drive**	0.001	1.6	-	-
**BIS/BAS Fun**	-0.006	1.7	-	-
**BIS/BAS Reward**	-0.007	1.7	-	-
**BIS/BAS Behavioral Inhibition**	0.004	1.8	-	-
**ERQ Reappraisal**	0.009	1.3	**0.160***	1.1
**ERQ Suppression**	-0.001	1.2	-	-
**WPI Extrinsic Motivation**	**-0.040****	2.6	**-0.374*****	1.6
**WPI Intrinsic Motivation**	0.019	2.0	-	-
**IMI Enjoyment**	0.001	1.9	-	-
**IMI Competence**	0.002	1.6	-	-
**IMI Effort**	-0.004	2.0	-	-
**IMI Tension**	0.001	1.5	-	-
**Adjusted R^2**	0.045		.116	

S2. Lottery Parameters

Following the conclusion of the surprise retest, we conducted a lottery for each participant. Participants earned a "lottery ticket" for each reward they had received during the task. The program generated random numbers (ranging from 1-800) representing each lottery ticket for each participant in a given lottery and then generated numbers for each of the cash prizes ($5, $15, and $20). If any of the numbers assigned to a lottery ticket matched one of the prize-winning numbers, the participant holding that ticket was paid the total amount of the prize/s in addition to their hourly compensation.

The probability of matching at least one number was ~.017, two numbers was ~.01 and three numbers was ~.0002.

S3. Participant Response Spelling Check

Once the participant had submitted their response for a given question, the testing program performed a spelling check on the response (based on the Enchant Spell Checker; Lachowicz, 2010) and suggested alternative spellings if the word was misspelled. In addition, in some cases, the participant might provide a word that was not misspelled, but was similar to, though not exactly, the answer in the database (e.g., "butterflies", when "butterfly" was the answer in the database; "Heracles", when "Hercules" was the answer in the database). The program was trained to identify these similar answers using a stored bank of "equivalents," and provide the database answer as an alternative. This was done to try to homogenize data output and ensure that answers were correctly categorized as reward-eligible or not. If alternatives were suggested, the participant had the option to select one of these alternatives, keep their original answer, or go back and retype a new response. If the subject chose the corrected spelling or the base answer from the alternatives and this item was correct and/or reward-eligible in the database, the program would categorize it accordingly. If they did not choose the alternative, the answer would not be considered reward-eligible and would be marked as incorrect.

S4. Group-Specific Instructions

Task instructions varied across the four study groups with respect to the nature of the rewards. In all groups, the instructions stated, "*These symbols have nothing to do with the accuracy of your answer, and you will see them appear after both incorrect and correct responses*." This statement was followed either by "*Rather, they are part of a separate and unrelated task*" (**Control group**) or "*Rather, it signifies whether or not you have gained a lottery ticket for that trial*" (**Reward groups**).

The instructions continued with:
Control group: "*In this task, your job is to mentally keep a running count of all the Yellow Disks/Circles that you see in each round of questions…. At the conclusion of each round you will report the number of Yellow Disks/Circles you counted. The closer your answer is to the actual number of Yellow Disks/Circles displayed, the more lottery tickets you will be given for your participation.*"

Award group: *Every time you see a Golden Ring/Coin it means that a number assigned to the question matches a* ***number randomly generated*** *by the computer.*

Luck group: *"Every time you see a Golden Ring/Coin it means that the computer has randomly drawn one of your "**lucky numbers**" that you will select in a few moments.*

Effort group: *Every time you see a Golden Ring/Coin it was deemed that you have made a sincere effort to provide a* ***good quality*** *answer.*

All Rewards: *Therefore, you will receive a lottery ticket for that trial, regardless of whether your answer was correct or not. If you see a Yellow Disk/Coin, it means that no lottery ticket was awarded.*

The instructions also varied slightly in the explanation of reward eligibility:

Control group: *"The computer generates the **Yellow Disk/Open Yellow Circle** randomly and relatively infrequently. However, the random generation process only occurs for questions that exceed a certain level of difficulty; in other words, counting-eligible questions are ones that are not too easy. So, although seeing a Yellow Disk/Open Yellow Circle has **nothing** to do with your performance on that trial, better **overall** performance will lead to you receiving more difficult questions, and therefore, to a greater likelihood of activating the random number generator and seeing a Yellow Disk/Open Yellow Circle that can earn you a lottery ticket if you count it correctly."*

Award group: *"The computer generates the **Golden Coin/Golden Ring** relatively infrequently. However, the random number generator is conducted only for questions that exceed a certain level of difficulty; in other words, award-eligible questions are ones that are not too easy.*
*So, although seeing a Golden Coin/Golden Ring has **nothing** to do with your performance on that trial, better **overall** performance will lead to you receiving more difficult questions, and therefore, a greater likelihood of activating the random number generator and receiving an award."*

Luck group: *"The computer generates the **Golden Coin/Golden Ring** relatively infrequently. However, the random drawing is conducted only for questions that exceed a certain level of difficulty; in other words, drawing-eligible questions are ones that are not too easy.*
*So, although seeing a Golden Coin/Golden Ring has **nothing** to do with your performance on that trial, better **overall** performance will lead to you receiving more difficult questions, and therefore, a greater likelihood of activating the random number generator and receiving an award."*

Effort group: *"As shown in the sample slides you saw moments ago, quality answers have certain characteristics that distinguish them from lazy or non-responsive answers, and the computer will select (and de-select) for those characteristics. Furthermore, the computer will consider the quality of your answer only when the question itself exceeds a certain level of difficulty; in other words, effort-eligible questions are ones that are not too easy. So better overall performance will lead to you receiving more difficult questions, and therefore, a greater likelihood of the computer evaluating your response effort and you receiving a reward."*

S5. Post-block Reward Stimulus Count

Following each of the 4 blocks of 40 questions, participants provided subjective self-reports on several measures, including the number of reward/target stimuli they had seen in the previous block. Participants in all groups were asked to accurately report the number of rewards they had received/targets they had seen, however only the participants in the Control group were incentivized to maintain an accurate count of these symbols. Specifically, control participants were told that "*the closer your answer is to the actual number displayed, the more lottery tickets you will be given*." Requiring the participants in the Control group to maintain an accurate count of these stimuli was designed to ensure that the appearance of the symbol that designated a reward in the other groups still constituted a meaningful event in the Control group. Likewise, because the number of reward stimuli related directly to the probability of additional cash payouts for the other groups, we anticipated that there was a high probability that participants in the three reward groups would be maintaining their own running count of the rewards, despite not being extrinsically incentivized to do so. Thus, we reasoned that requiring Control participants to maintain an accurate count of rewards would help mimic the cognitive processes occurring within the other groups.

Nonetheless, given the explicit emphasis on maintaining a stimulus count, we predicted that the Control group would be more accurate in their actual stimulus count estimates. To test this hypothesis, we calculated the magnitude of the stimulus count error by taking the absolute difference

between the number of stimuli counted across the four study blocks and the total number of actual stimuli shown. This provided a measure of the counting error magnitude irrespective of direction (i.e. overestimation or underestimation). We then conducted an ANOVA on the counting error magnitude to determine whether count accuracy differed as a function of group or gender. Due to a scripting error, data was missing from one subject in the Effort group for all post-block questions. The analyses below include the remaining 139 participants. A two-way analysis of variance (ANOVA) on the counting error showed a main effect of group, $F(3, 131) = 3.72$, $p = .013$, $\eta p^2 = .078$. Overall, participants in the Control group ($M = 5.71$, $SD = 14.16$) kept a more accurate count of stimuli than any other group, although this difference only reached significance between participants in the Control and Award groups ($M = 15.06$, $SD = 13.68$, $p = .009$). The main effect of gender and the group by gender interaction were not significant, (all *F*s < 1.8, *p*s > .19, ηp^2s < .02.

To test whether counting error magnitudes significantly differed from zero, we conducted a series of one-sample t-tests (Bonferroni adjusted $p < .006$) for men and women in each group separately. The t-tests indicated that neither men nor women in the Control group had errors that were significantly different from zero, indicating that participants in this group provided relatively accurate counts of the reward stimulus feedback. By contrast, men and women in all other study groups had error scores that were significantly greater than zero.

Given that counting was more accurate in the Control group, one potential concern is that the effort of maintaining accurate stimulus counts resulted in greater cognitive load for the Control group, which in turn created group-level performance differences in encoding of the corrective feedback that were unrelated to the reward framing itself. If so, we would expect to find that more accurate stimulus counts in this group would relate to the magnitude of performance detriment on the two tests. However, correlations between the error of the reward stimulus count and either first-test or retest accuracy revealed the opposite pattern.

Collapsing across all four groups and two genders in order to maximize power, the stimulus counting error magnitude correlated negatively with performance on the first test ($r[137] = -.17$, $p = .05$), and marginally with the retest ($r[137] = -.15$, $p = .08$). Thus, participants who had fewer errors in the number of stimuli reported had slightly higher test accuracy, suggesting that maintaining an accurate count of the stimuli did not interfere with test performance. In order to confirm that this pattern was consistent across groups, we also ran separate correlations for each group and found a similar negative relationship across all 8 tests (see Table 2). However, the only correlation that reached significance was between counting error magnitude and first test accuracy in the Award group ($r[34] = -.35$, $p = .04$; uncorrected for multiple comparisons). The correlations between count and test accuracy were non-significant in the Control group, yet both were in the negative direction. From this we conclude that although Control group's instruction to maintain an accurate stimulus count did improve count accuracy, this did not come at the cost of test performance.

Table 2. Correlations between stimulus counting error magnitude with test accuracy

Group	N	w/First Test Accuracy (r)	p-val	w/Retest Accuracy (r)	p-val	Deg. of freedom
Control	35	0.00	0.99	-0.27	0.11	33
Effort	35	-0.04	0.83	-0.25	0.15	33
Luck	34	-0.26	0.14	-0.08	0.64	32
Award	36	-0.35	**0.04**	-0.12	0.49	34
All Subs	139	-0.17	**0.05**	-0.15	0.08	137

* Correlation is significant at the 0.05 level (2-tailed).

S6. First-Test Measures

Prior to conducting our primary analyses, we verified that first-test measures were equated across the four groups and for both genders. We conducted a series of analyses to test whether there were differences with respect to group or gender for first test performance, the delay interval between Day 1 and Day 2, the number of rewards received (total number of rewards, and the division of rewards across correct and error response trials), and the average confidence ratings for rewarded and non- rewarded trials.

First-test performance was calculated as the proportion of items initially correct (where the target accuracy for the titration algorithm was 0.50). We found no significant effects main effects or interactions (all *F*s < 1.5, *p*s > .5, ηp^2s < .05). These results indicate that our titration algorithm was effective in equating initial performance across groups and genders in this study.

We then conducted a Pearson chi-square test of independence to ensure that delay intervals did not systematically differ with respect to either group or gender. Results of the chi-square test were non-significant, indicating that the delay intervals were balanced across both group, $X^2(3, N = 140) = .704$, $p = .87$, and gender, $X^2(1, N = 140) = 1.65$, $p = .2$.

We also verified that the number of rewards administered at first test was equated across all other factors, including trial accuracy. A repeated measures ANOVA with trial accuracy (correct, incorrect) as a within-subjects factor and group and gender as the between-subjects factors found only a main effect of accuracy ($F[1, 132] = 84.16$, $p < .001$, $\eta_p^2 = .389$). Despite the testing program's algorithm for balancing the rewards between correct and incorrect trials, participants nonetheless received significantly more rewards following correct (M = 23.93, SD = .285) than incorrect responses (*M* = 22.84, *SD* = 1.3), regardless of group or gender. However, the mean difference between these two accuracy types was quite small (just over 1 reward on average). Likewise, as both rewards for correct and incorrect responses were equally balanced across factors of gender and group, this difference should not confound interpretation of any effects of those between-subjects variables.

As a precautionary measure, we also compared the mean confidence levels across rewarded and non-rewarded trial types to ensure that there were no systemic differences between them other than the presence of the reward feedback. We found no significant main effects or interactions (all *F*s < .7, *p*s > .3, η_p^2s < .02). Thus, there were no apparent differences in confidence between rewarded and non-rewarded trials across either group or gender.

Table 1S. First-Test Measures. Standard errors of the mean appear in parentheses.

	**Men**				**Women**			
	**Control**	**Effort**	**Luck**	**Award**	**Control**	**Effort**	**Luck**	**Award**
**First Test Accuracy**	0.5026 (0.003)	0.5017 (0.003)	0.4979 (0.003)	0.4969 (0.005)	0.4967 (0.003)	0.5028 (0.002)	0.497 (0.003)	0.4986 (0.002)
**Delay (hours)**	28.79 (3.35)	32.35 (3.28)	35.96 (3.18)	30.81 (3.93)	31.14 (2.60)	29.98 (2.78)	27.81 (2.62)	31.68 (2.33)
**Total Number**	46.67 (0.47)	47.13 (0.32)	46.33 (0.44)	47.10 (0.35)	46.61 (0.29)	47.15 (0.24)	46.47 (0.35)	46.81 (0.24)

Rewards

**Number Correct Rewarded**	23.92 (0.08)	24 (0.00)	23.93 (0.07)	23.9 (0.10)	23.96 (0.04)	23.95 (0.05)	23.79 (0.12)	23.96 (0.04)
**Number Errors Rewarded**	22.75 (0.45)	23.13 (0.32)	22.4 (0.46)	23.2 (0.36)	22.65 (0.29)	23.2 (0.21)	22.68 (0.29)	22.85 (0.24)
**Mean Confidence Rewarded**	3.42 (0.13)	3.57 (0.15)	3.50 (0.16)	3.49 (0.22)	3.09 (0.13)	3.44 (0.16)	3.34 (0.12)	3.32 (0.15)
**Mean Confidence No Reward**	3.46 (0.17)	3.60 (0.16)	3.45 (0.16)	3.45 (0.29)	3.14 (0.12)	3.36 (0.17)	3.19 (0.15)	3.41 (0.14)

S7. Metacognitive Sensitivity

Metacognitive sensitivity refers to the relationship between the participant's confidence and the accuracy of their response. High sensitivity is indicative of a tight correspondence between accuracy and confidence in which accurate responses tend to be endorsed with higher confidence and vice versa. Applying a Signal Detection Theory approach (Green & Swets, 1966), we examined whether sensitivity differed across groups and gender or as a function of time (i.e., first test compared to retest). Following the methods set forth in Fleming & Lau (2014), we calculated an AUROC (area under receiver operator characteristic) metric for each participant for both the first test and the retest[1]. AUROC is a measure of sensitivity that is unaffected by the participant's response bias (i.e., a tendency to cluster ratings around the upper or lower boundaries of the confidence scale). We then compared sensitivity using a repeated-measures ANOVA with time as the within-subjects factor, and group and gender as between-subject factors. The analysis revealed a significant main effect of time, $F(1, 132) = 291$, $p < .001$, $\eta p^2 = .688$. Participants had greater sensitivity during the retest ($M = .90$, $SD = .05$) than during the first test ($M = .82$, $SD = .04$). Given that participants were provided with both accuracy feedback as well as the correct answer following each question on the first test, this finding was expected. We also found a main effect of gender, $F(1, 132) = 5.81$, $p = .017$). Women ($M = .87$, $SD = .04$) had higher sensitivity overall as compared to men ($M = .85$, $SD = .02$), however no other main effects or interactions were significant (all Fs < 1.8, ps > .17, ηp^2s < .5).

[1] AUROC was calculated using a freely available Matlab script that can be found in the supplemental section of (Fleming & Lau, 2015).

References

Amabile, T. M., Hill, K. G., Hennessey, B. A., & Tighe, E. M. (1994). The Work Preference Inventory: assessing intrinsic and extrinsic motivational orientations. *Journal of Personality and Social Psychology*, *66*(5), 950–967.

Carver, C. S., & White, T. L. (1994). Behavioral inhibition, behavioral activation, and affective responses to impending reward and punishment: the BIS/BAS scales. *Journal of Personality and Social Psychology*, *67*(2), 319-333.

Crawford, J. R., & Henry, J. D. (2004). The Positive and Negative Affect Schedule (PANAS): Construct validity, measurement properties and normative data in a large non-clinical sample. *British Journal of Clinical Psychology*, *43*(3), 245–265.

Elliot, A. J., & Murayama, K. (2008). On the measurement of achievement goals: Critique, illustration, and application. *Journal of Educational Psychology*, *100*(3), 613-628.

Green, D. M. & Swets, J. A. (1966). Signal detection theory and psychophysics. Oxford, England: John Wiley.

Gross, J. J., & John, O. P. (2003). Individual differences in two emotion regulation processes: implications for affect, relationships, and well-being. *Journal of Personality and Social Psychology*, *85*(2), 348-362.

Fleming, S. M. & Lau, H. C. (2014). How to measure metacognition. Frontiers in Human Neuroscience, 8, Article 443.

Lachowicz, D. (2010). Enchant Spell Checker. AbiWord. Retrieved from http://abisource.com/projects/enchant/

Ryan, R. M. (1982). Control and information in the intrapersonal sphere: An extension of cognitive evaluation theory. *Journal of Personality and Social Psychology, 43(3)*, 450-461.
